# Resolving Artifacts in Voltage‐Clamp Experiments with Computational Modeling: An Application to Fast Sodium Current Recordings

**DOI:** 10.1002/advs.202500691

**Published:** 2025-06-06

**Authors:** Chon Lok Lei, Alexander P. Clark, Michael Clerx, Siyu Wei, Meye Bloothooft, Teun P. de Boer, David J. Christini, Trine Krogh‐Madsen, Gary R. Mirams

**Affiliations:** ^1^ Institute of Translational Medicine, Faculty of Health Sciences University of Macau Macau 999078 China; ^2^ Department of Biomedical Sciences, Faculty of Health Sciences University of Macau Macau 999078 China; ^3^ Department of Biomedical Engineering Cornell University Ithaca NY 14853 USA; ^4^ Centre for Mathematical Medicine & Biology, School of Mathematical Sciences University of Nottingham Nottingham Nottinghamshire NG7 2RD UK; ^5^ Department of Physiology & Pharmacology SUNY Downstate Health Sciences University Brooklyn NY 11203 USA; ^6^ Department of Medical Physiology, Division of Heart and Lungs University Medical Center Utrecht Utrecht 3584 CM Netherlands; ^7^ Department of Physiology & Biophysics Weill Cornell Medicine New York NY 10065 USA; ^8^ Institute for Computational Biomedicine Weill Cornell Medicine New York NY 10065 USA

**Keywords:** computational model, experimental error, fast sodium current, patch clamp, voltage clamp

## Abstract

Cellular electrophysiology underpins fields from basic science in neurology, cardiology, and oncology to safety critical applications for drug safety testing, risk assessment of rare mutations, and models based on cellular electrophysiology data even guide clinical interventions. Patch‐clamp voltage clamp is the gold standard for measuring ionic current dynamics that explain cellular electrophysiology, but recordings can be influenced by artifacts introduced by the measurement process. A computational approach is developed, validated through electrical model cell experiments, to explain and predict intricate artifacts in voltage‐clamp experiments. Applied to various cardiac fast sodium current measurements, the model resolved artifacts in the experiments by coupling observed current with simulated membrane voltage, explaining some typically observed shifts and delays in recorded currents. It is shown that averaging data for current‐voltage relationships can introduce biases comparable to effect sizes reported for disease‐causing mutations. The computational pipeline provides improved assessment and interpretation of voltage‐clamp experiments, correcting, and enhancing understanding of ion channel behavior.

## Graphical abstract

Patch‐clamp experiments are fundamental to the measurement and understanding of ion currents that underlie physiological functions from neural activity to muscle contraction. Via a mathematical model of the experimental apparatus, this article highlights how artifact effects can be as large as some reported mutation and drug effects, and enables computational models to allow for artifacts when calibrating to experimental data.

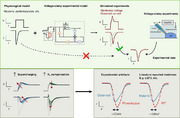

## 1 Introduction

The pioneering work of Hodgkin & Huxley^[^ ^1^, ^2^ ^]^ provided fundamental insights into cellular excitability, marking the start of the modern era of systems electrophysiology research. This field has provided deep understanding of diverse biological processes in the areas of neurology,^[^ ^3^ ^]^ cardiology,^[^ ^4^ ^]^ oncology,^[^ ^5^ ^]^ safety‐critical applications for drug safety testing,^[^ ^6^ ^]^ and many more. Patch‐clamp experiments have been the gold standard for the study of cellular electrophysiology since their invention.^[^ ^7^ ^]^ In particular, whole‐cell mode voltage clamp allows us to probe the dynamic current responses of an electrically excitable cell while controlling its voltage. Measured whole‐cell current is typically carried by a collection of transmembrane ion‐permeable proteins, each with distinct opening and closing kinetics (often nonlinear) that are studied through voltage clamp experiments. These studies and applications rely on the assumption that membrane voltage is clamped precisely to desired voltages. Here, we demonstrate when and how this assumption breaks down, and provide strategies to improve interpretation of imperfect voltage‐clamp data.

Imperfect voltage clamping is pervasive in electrophysiology research and contributes to experimental artifacts that can cause incorrect characterization of channel and cell properties. Errors primarily arise due to cell and pipette capacitances, leak current, and series resistance between the pipette electrode and cell membrane.^[^ ^8^ ^]^

In post‐processing, Marty and Neher^[^ ^8^ ^]^ suggested a correction to current‐voltage (*I*–*V*) summary curves: using estimates of the series resistance to predict voltage drop for a given current, and then to translate each point horizontally on the *I*–*V* curve appropriately. But this approach does not account for all the artifacts we know to be present in the system, or correct for the loss of voltage clamp that can occur during an experiment. Modern amplifiers also have compensatory mechanisms designed to partially correct for the unwanted effects of artifacts during acquisition. Cell capacitance is compensated for using a mechanism that speeds membrane charging.^[^ ^9^, ^10^ ^]^ Amplifiers also compensate for artifacts that result from series resistance: namely, the slowing of the membrane voltage's approach to the command voltage,^[^ ^10^, ^11^ ^]^ and the deviation of the membrane voltage from the command voltage.^[^ ^12^, ^13^, ^14^, ^15^ ^]^

These strategies, however, still rely on imperfect estimates of series resistance and capacitance, and create a system that is sensitive to overcompensation, with unphysiological dynamics. Thus, whole cell patch‐clamp artifacts remain an issue, especially when measuring currents with fast dynamics, such as sodium ion currents.

Computational adjustment for incomplete series resistance compensation was proposed in the 1990s.^[^ ^16^ ^]^ In previous work, we showed that a series‐resistance compensation model could explain variation in potassium channel data.^[^ ^17^ ^]^ Montnach et al.^[^ ^18^ ^]^ showed how a similar approach improved interpretation of fast sodium dynamics. Lei^[^ ^19^ ^]^ developed a computational model of the amplifier dynamics compensating for the slow approach of membrane voltage to the command voltage — a compensation technique called *prediction* or *supercharging*. Abrasheva et al.^[^ ^20^ ^]^ showed how including supercharging improves mathematical model fits to fast sodium data, however potentially failing to correct steady state effects due to missing series resistance compensation in that model.

**Figure 1:**
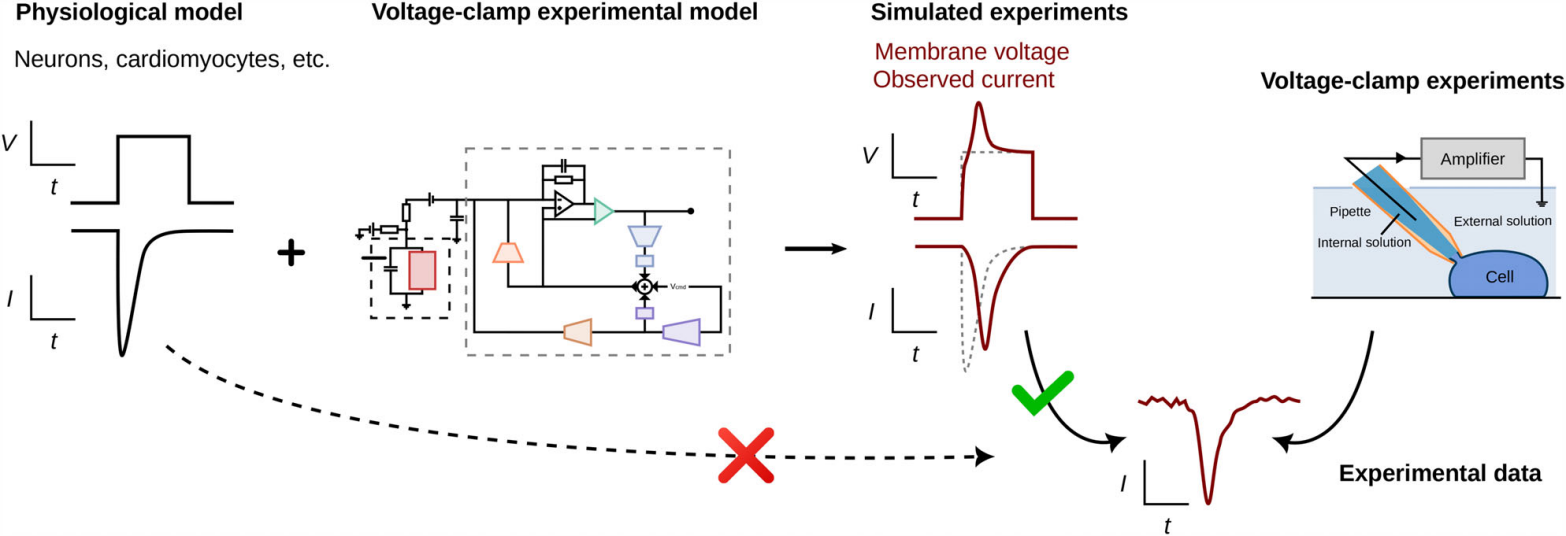
A workflow schematic comparing our approach, which uses realistic simulations of the voltage‐clamp process, to the standard approach in which overly‐idealized assumptions are made about experimental data quality. Our mathematical model of patch‐clamp voltage clamp produces simulated experimental data that can be more accurately compared to experimental data that are affected by patch‐clamp artifacts.

In this study, we develop a new computational approach that allows us to better explain and predict artifacts during voltage‐clamp experiments to further improve interpretation of sodium current recordings (**Figure** **1**). The computational model includes equations that reproduce the majority of voltage‐clamp artifacts and the compensatory circuits in amplifiers (e.g., capacitance / series resistance compensation, supercharging, and liquid junction potential / voltage offset correction), including separate parameters for estimated and true values of series resistance and cell capacitance. We validate and test the model on sodium current at physiological temperature, with multiple patching strategies / cell types, and several compensation levels to demonstrate the artifact model's ability to reproduce dynamics under extreme and varying conditions. We show the ability of this computational model to reproduce artifact‐causing dynamics in an electrical model cell, in human induced pluripotent stem cell‐derived cardiomyocytes (hiPSC‐CMs), and in Na_V_1.5‐expressing human embryonic kidney (HEK) cells. The model improves the interpretation of data recorded with different amplifiers, using rupture or perforated patch, and both manual and automated patch techniques. We believe this model represents a step forward in how voltage‐clamp data can be interpreted. Specifically, we (1) provide a tool to improve interpretation of electrophysiological recordings, and (2) make it possible to obtain accurate estimates of biophysical properties that were previously obscured by experimental artifacts.

## 2 Results

### 2.1 A Detailed Computational Model for Patch‐Clamp Voltage‐Clamp Experiments

**Figure 2:**
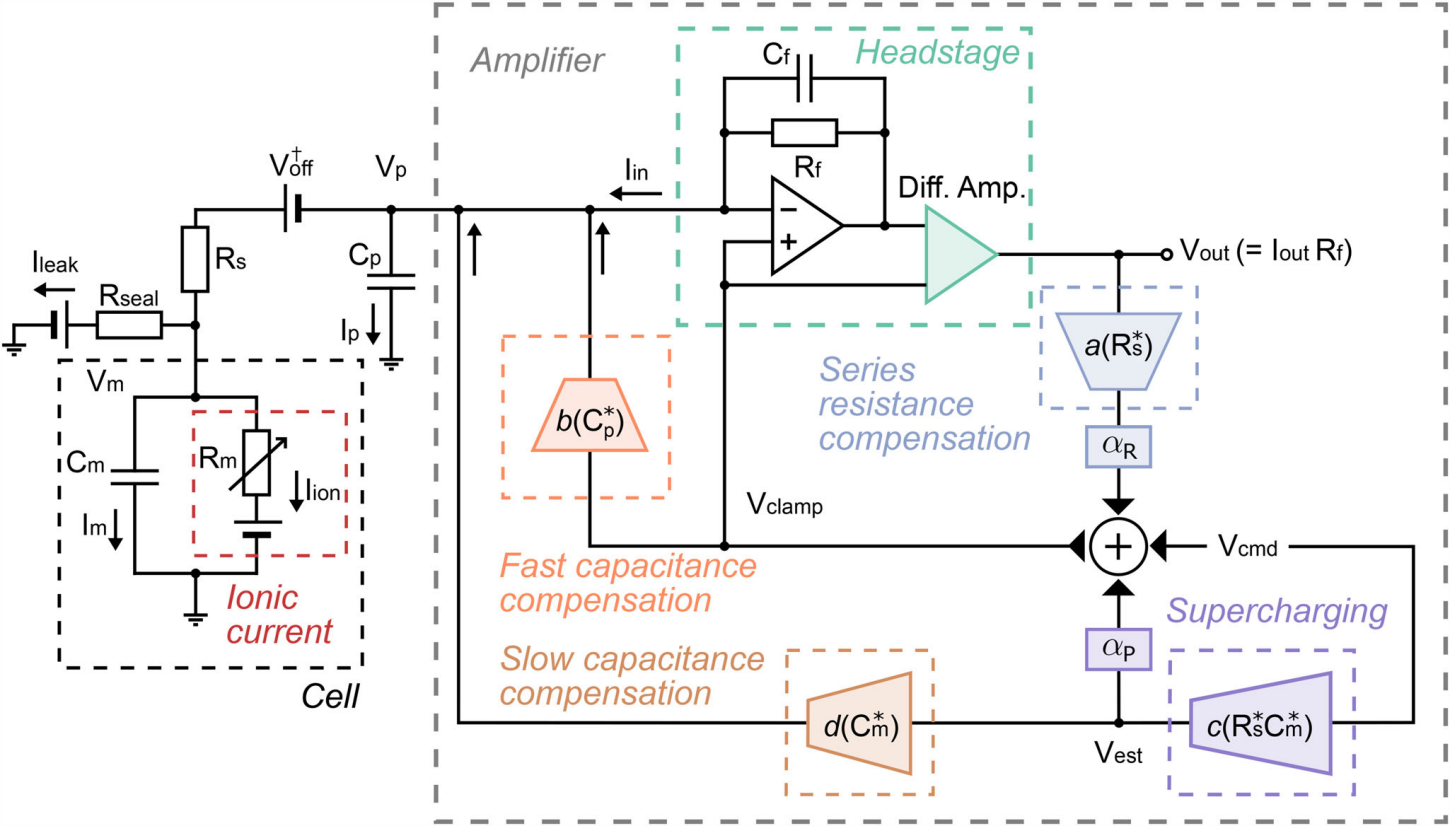
A detailed schematic of the equivalent electrical circuit of the new detailed mathematical model for whole‐cell patch‐clamp voltage‐clamp experiments. Right shows the components of an patch‐clamp amplifier, connecting to the cell on the left. The amplifier includes the components for compensating for series resistance, supercharging, slow capacitance, fast capacitance, and liquid junction potential and voltage offset. The leak correction is modeled as a post‐processing step that is not part of the equivalent circuit.

We developed a model that quantitatively, comprehensively describes the detailed dynamic interaction between all voltages and currents in a patch‐clamp voltage‐clamp experiment, including the amplifier compensation circuitry (**Figure** **2**). The first version of our mathematical model of voltage‐clamp experimental artifacts was published in Lei et al.^[^ ^17^ ^]^ that described only the basic effects of series resistance, pipette capacitance, cell membrane capacitance, and leak current in the experiment, as well as some of their compensation such as series resistance compensation. Here we expand it to include all the essential functionality of a modern voltage‐clamp amplifier, including a new supercharging component (purple component in Figure 2), and derive its interaction with series resistance compensation (blue component in Figure 2). With supercharging compensation, an amplifier will estimate (based on an estimate of cell capacitance *C*_m_ and series resistance *R*_s_) and inject the current required to ‘over‐charge’ the cell membrane to the desired voltage level (i.e., the command voltage). Overcompensation in electrophysiological studies can cause harm to cells and deleterious artifact — thus, compensation is often scaled down to 70 % to 80 % of the estimated value of full compensation. The level of compensation is controlled by the parameter α_P_ (Equation (5)), with *zero* being no compensation and *one* being full compensation. In the next few sections, we examine the effect of this parameter, along with series resistance compensation (α_R_, Equation (6)).

The formulation of the new supercharging component of our model was first introduced in the first‐author's PhD thesis^[^ ^19^ ^]^ and later independently reported in Abrasheva et al.^[^ ^20^ ^]^ The amplifier and compensation delays have been further updated in this new version based on the amplifier settings, as validated in the next section.

Our model puts together the new components and the fast / slow capacitance compensations (orange and brown components in Figure 2) corresponding to the compensations for the current induced by the pipette capacitance *C*_p_ and cell membrane capacitance *C*_m_, liquid junction potential and voltage offset *V*_off_ compensation, and leak current *I*_leak_ correction. Aggregating all these components (Figure 2), the updated model can be expressed as follows:

(1) $$I_{\mathrm{ion}}=f\left(t,V_{m}\right)\;\text{Ion channel current}$$

(2) $$I_{\mathrm{leak}}=g_{\mathrm{leak}}\left(V_{m}-E_{\mathrm{leak}}\right)\;\text{Leak current}$$

(3) $$\begin{array}{ccc} \frac{dV_{m}}{dt} & = & \;\frac{1}{R_{s}C_{m}}\left(V_{p}+V_{\mathrm{off}}^{†}-V_{m}\right)\;V_{\mathrm{off}}\;\text{compensation}\\ & & -\frac{1}{C_{m}}\left(I_{\mathrm{ion}}+I_{\mathrm{leak}}\right) \end{array}$$

(4) $$\frac{dV_{p}}{dt}=\frac{1}{\tau_{\mathrm{clamp}}}\left(V_{\mathrm{clamp}}-V_{p}\right)\;\text{Amplifier delay}$$

(5) $$\frac{dV_{\mathrm{est}}}{dt}=\frac{V_{\mathrm{cmd}}-V_{\mathrm{est}}}{\left(1-\alpha_{P}\right)R_{s}^{*}C_{m}^{*}}\;\text{Supercharging}$$

(6) $$V_{\mathrm{cmd}}^{\prime }=V_{\mathrm{cmd}}+R_{s}^{*}\left(\alpha_{R}I_{\mathrm{out}}+\alpha_{P}C_{m}^{*}\frac{dV_{\mathrm{est}}}{dt}\right)\;R_{s}\;\text{compensation}$$

(7) $$\frac{dV_{\mathrm{clamp}}}{dt}=\frac{1}{\tau_{\mathrm{sum}}}\left(V_{\mathrm{cmd}}^{\prime }-V_{\mathrm{clamp}}\right)\;\text{Compensation delay}$$

(8) $$\begin{array}{cccc} I_{\mathrm{in}}= & \;I_{\mathrm{ion}}+I_{\mathrm{leak}} & & \\ & +C_{p}\frac{dV_{p}}{dt}-C_{p}^{*}\frac{dV_{\mathrm{clamp}}}{dt} & & C_{p}\;\text{compensation}\\ & +C_{m}\frac{dV_{m}}{dt}-C_{m}^{*}\frac{dV_{\mathrm{est}}}{dt} & & C_{m}\;\text{compensation} \end{array}$$

(9) $$\begin{array}{cccc} \frac{dI_{\mathrm{out}}}{dt}= & \;\frac{1}{\tau_{z}}\left(I_{\mathrm{in}}-I_{\mathrm{out}}\right) & & \mathrm{Obse}\mathrm{rved}\mathrm{curr}\mathrm{ent} \end{array}$$

(10) $$\begin{array}{cccc} I_{\mathrm{post}}= & \;I_{\mathrm{out}}-g_{\mathrm{leak}}^{*}\left(V_{\mathrm{cmd}}-E_{\mathrm{leak}}^{*}\right) & & \text{Post-processed}\;\text{current} \end{array}$$

Note that all the derivatives appearing on the right‐hand side can be rewritten in terms of state variables, but they appear for concise notation. Machine or post‐processing estimates of parameters *X* denoted as X*, and error in estimates as *X*†. For an explanation of the other symbols and parameters see Supporting Information. We refer to this set of equations as the voltage‐clamp model.

### 2.2 Experimental Validation of the Voltage‐Clamp Model

**Figure 3:**
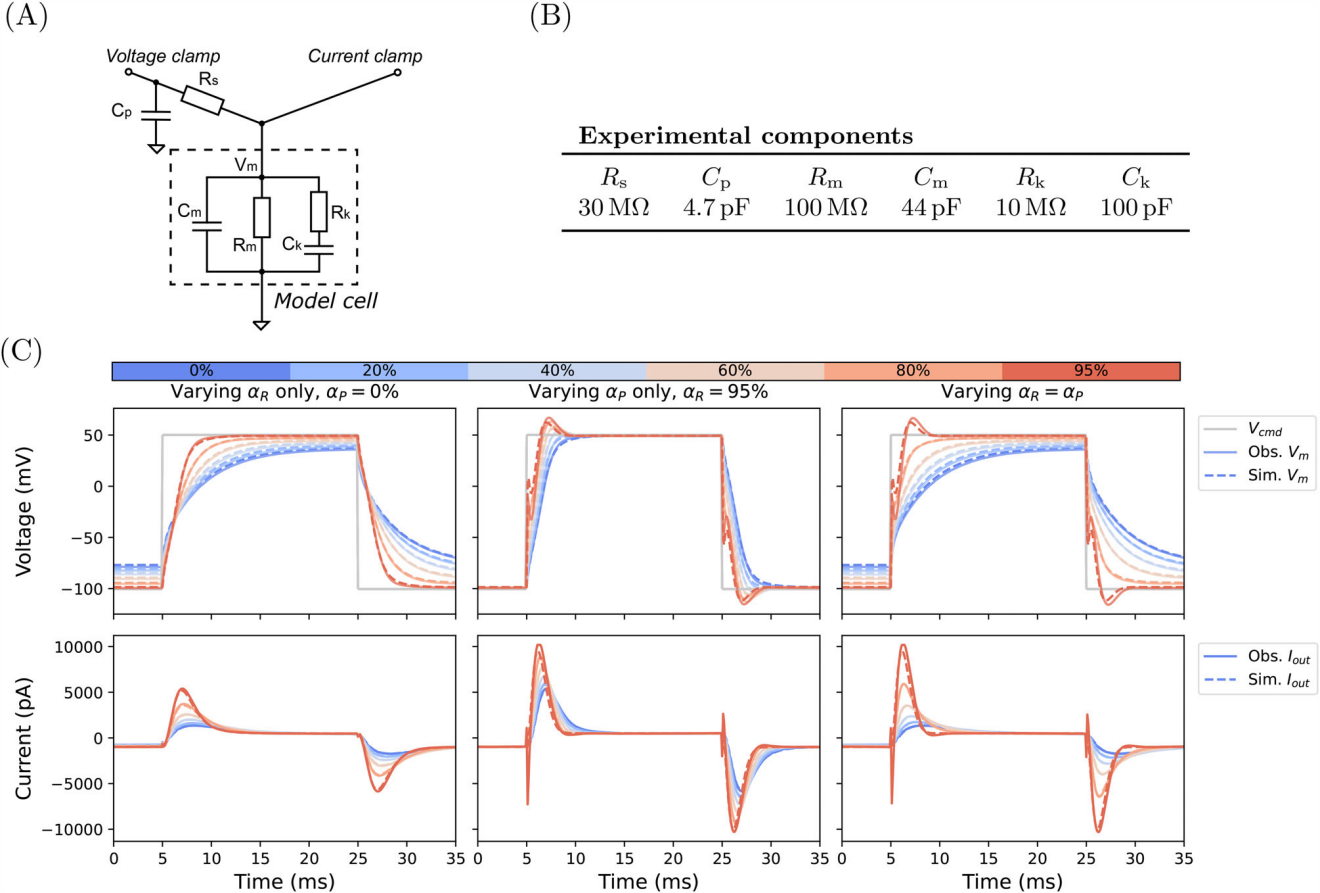
Experiments to validate our mathematical model of patch clamp artifacts and compensations using a physical electrical model cell. A) A schematic of the electrical model cell (a modification of the ‘Type II’ model cell used in Lei et al.^[^ ^17^ ^]^ with faster dynamics). B) The chosen nominal component values. C) Solid lines are experimentally measured, and dashed lines are simulated model outputs. (top) membrane voltage *V*_m_ (gray line is command voltage *V*_cmd_), and (bottom) output current *I*_out_ at different levels of compensation. Left: effect of series‐resistance compensation (α_*R*_) alone. Middle: supercharging compensation (α_*P*_) alone. Right: varying both compensation levels together (α_*R*_ = α_*P*_). The electrical circuit produces indistinguishable replications.

Before testing the descriptive capacity of the voltage‐clamp model in a biological system, where parameter values can only be approximated, we first validated the equations using a tailor‐made hardware circuit. This circuit included electrical components with known physical values. It was connected to a voltage‐clamp amplifier, as one would connect a biological cell (**Figure** **3**A, labeled as “voltage clamp”). Part of the hardware circuit contained an electrical “model cell” that was designed with multiple resistor‐capacitor (RC) circuits in series and in parallel to elicit responses similar to a biological cell, which resulted in dynamics mimicking the effects of cell capacitance, membrane resistance, and a fast ionic current.

A second amplifier was used to directly measure the membrane voltage of the electrical model cell in current‐clamp mode with zero current injection (labeled as “current clamp” in Figure 3A). This extra amplifier was required as one amplifier cannot simultaneously set the command voltage (through *R*_s_) and measure the membrane voltage (bypassing *R*_s_). The electrical component values of the hardware circuit shown in Figure 3B were chosen to mimic the size and rapid gating of fast ionic current recordings, and we derive equations for its ‘ionic current’ (*I*_ion_) in Supporting Information.

To test the voltage‐clamp equations, we compared the dynamics when systematically adjusting the series resistance and supercharging compensation parameters ($\alpha_{R}$ and $\alpha_{P}$) in the hardware circuit and mathematical voltage‐clamp model. We chose to adjust only these parameters, as all other parameters were known, and these are commonly adjusted parameters during biological experiments. When performing experiments with the electrical model cell, we went beyond the typically recommended maximum series resistance of around 80 %, and stress tested the model by pushing the compensation to a maximum of 95 %. We note that such a high level of compensation is not generally recommended for biological cell experiments, as it can lead to oscillations of potential that degrades data quality and may affect cell viability. A simple large voltage step from −100 mV to 50 mV for 20 ms was used as the command voltage. This voltage step should elicit relatively large currents from the electrical model cell, mimicking the current and artifact sizes seen in fast sodium current recordings. With these choices, the electrical model cell experiments provide a strong ‘stress‐test’ for the mathematical model.

Figure 3C shows the results of testing three types of compensation settings: For the first column (Figure 3C) the supercharging (prediction, α_*P*_) was fixed to zero and series resistance compensation (α_*R*_) was varied from 0 % (no compensation) to 80 % with an increment of 20 %, and finally set to 95 %. In the second column (Figure 3C), we tested with α_*R*_ was set to the maximum (95 %) and α_*P*_ varied from 0 % to its maximum. In the third column (Figure 3C), we tested varying α_*R*_ and α_*P*_ together, as most commonly used during biological experiments. The mathematical model was able to predict the experimental results excellently for compensation levels up to 80 %, with differences from experiment generally not visible by eye. For 95 % compensation of both series resistance and prediction, although the simulations do become distinguishable from the measurements, the qualitative shapes of the responses are correct; with complex deflections, overshoots and turning points all predicted well. These results demonstrate that with a model for *I*_ion_ with known parameters we are able to predict experimental errors and effects of amplifier compensations with high accuracy.

### 2.3 Computational Model Predicts Compensation Effects on in vitro Fast Sodium Current Measurements

**Figure 4:**
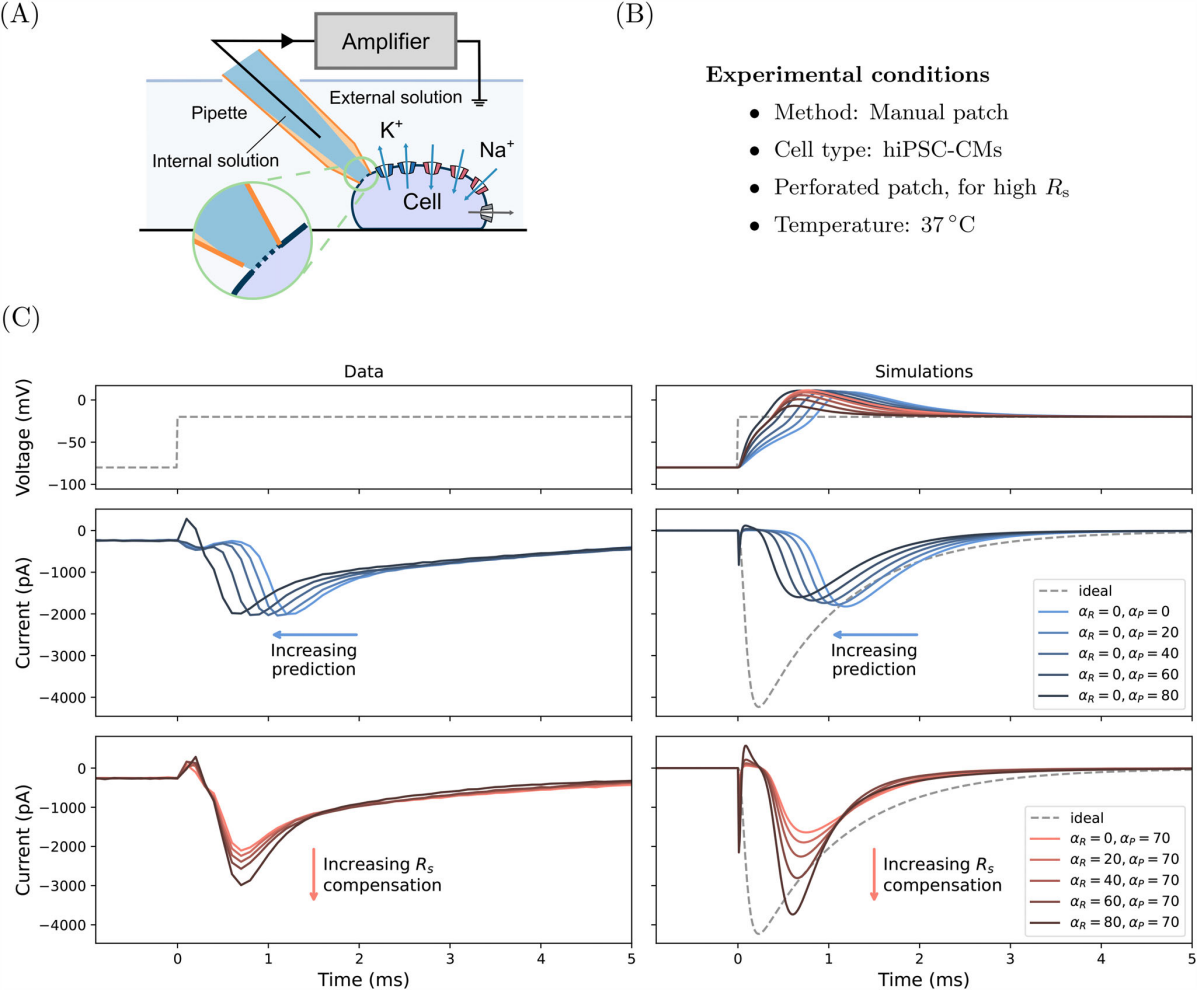
Assessing fast sodium current responses to experimental artifacts, with experimental recordings in hiPSC‐CMs. A) A schematic of the experimental setup and B) the experimental conditions for the perforated patch experiments with hiPSC‐CMs to achieve high series resistance. C) Left: Patch‐clamp voltage‐clamp in vitro experimental data measured from hiPSC‐CMs, with estimated *R*_s_ = 19.5 MΩ and *C*_m_ = 27.4 pF (*n* = 4, other repeats are shown in Supporting Information); Right: Simulations of the voltage‐clamp model (Equation (1)–(10)) using the Paci et al.^[^ ^21^ ^]^ hiPSC‐CM model for *I*_ion_ (including all of its ion currents but with traces here dominated by the Na_V_1.5 current). From top to bottom, it shows the command voltage (dashed lines) and the membrane voltage (solid lines), varying only the level of prediction (α_*P*_, blue), and varying only the level of series resistance compensation (α_*R*_, red), showing distinctive effects between the two. Note how the simulations reveal that the results are explained by a large loss of voltage clamp (top right panel of C), but the data can still be interpreted via the mathematical model.

We next explored whether the computational voltage‐clamp model could predict the effect of changes in compensation levels on experimental recordings of the fast sodium current in vitro. We tested the model's ability to reproduce the dynamics of sodium current elicited from hiPSC‐CMs in a perforated patch setup at 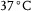 (as shown schematically in **Figure** **4**A and B, with further details given in Experimental Section). Perforated patch results in a large series resistance between the pipette and cell cytoplasm, exacerbating the experimental artifact effects on patch‐clamp recordings.

Fast sodium currents were elicited by applying a single voltage step (Figure 4C, left). In this setting artifacts can distort the applied voltages and recorded currents. Figure 4C (right) shows our mathematical voltage‐clamp model when applied to a fast sodium current model from an hiPSC‐CM cell model of electrophysiology.^[^ ^21^ ^]^ We also show the ‘ideal’ voltage clamp (gray, dashed) with the same fast sodium current model but no experimental artifacts. As before, compensation levels were varied to assess the overall performance.

First, the different responses to the prediction levels (α_*P*_, blue) were examined without series resistance compensation (α_*R*_ set to 0). The current was left‐shifted (peaks occurred earlier) as the level of prediction / supercharging was increased; this phenomenon was captured by the new voltage‐clamp model, which even predicted the small ‘hump’ in recorded current at the beginning of the voltage step. The leftward shift can be explained by looking at the simulated membrane voltage (top panel) which changes due to a ‘loss of voltage clamp’; increasing prediction reduces the membrane charging time, thereby reducing the time to reach the command voltage and thus reducing the time until peak current is reached (middle row).

Next, the responses to the series resistance compensation were compared (α_*R*_, red) with α_*P*_ = 80 %. In this case, instead of correcting the delay of the measured current, series resistance compensation improves its amplitude by bringing the membrane voltage closer to the command voltage. At the highest compensation level, α_*R*_ = α_*P*_ = 80 %, the measured current (dark red) was much closer to the ideal current (black dashed line). However, even that was not enough to obtain the desired voltage step (as shown by the simulation) which can result in erroneous estimates of the underlying physiological characteristics, as demonstrated in the next section. Repeats of the experiments are shown in Supporting Information.

Note how the delay in reaching the command voltage was about 3 ms at most, which is why prediction (or supercharging) is particularly important for currents with very rapid kinetics. Both effects are exacerbated when current magnitudes are larger, which is why both types of compensation are important for large magnitude currents such as fast sodium ion currents.

### 2.4 Extrapolating Physiological *I*–*V* Relationships from Imperfect Data

Next, we demonstrate how to use our computational model to extrapolate from current–voltage (*I*–*V*) relationships based on imperfect data (even under the best experimentally possible conditions) to the potential underlying physiological *I*–*V* relationships, leading to an improved estimate of maximum current conductance. In this approach, we fit the voltage‐clamp model and a mathematical model of the fast sodium current^[^ ^22^ ^]^ to experimental data (with 80% series resistance compensation and prediction or supercharging), by changing model parameters *R*_s_, *C*_m_, $V_{\mathrm{off}}^{†}$, and the maximum conductance parameter of the fast sodium model.

**Figure 5:**
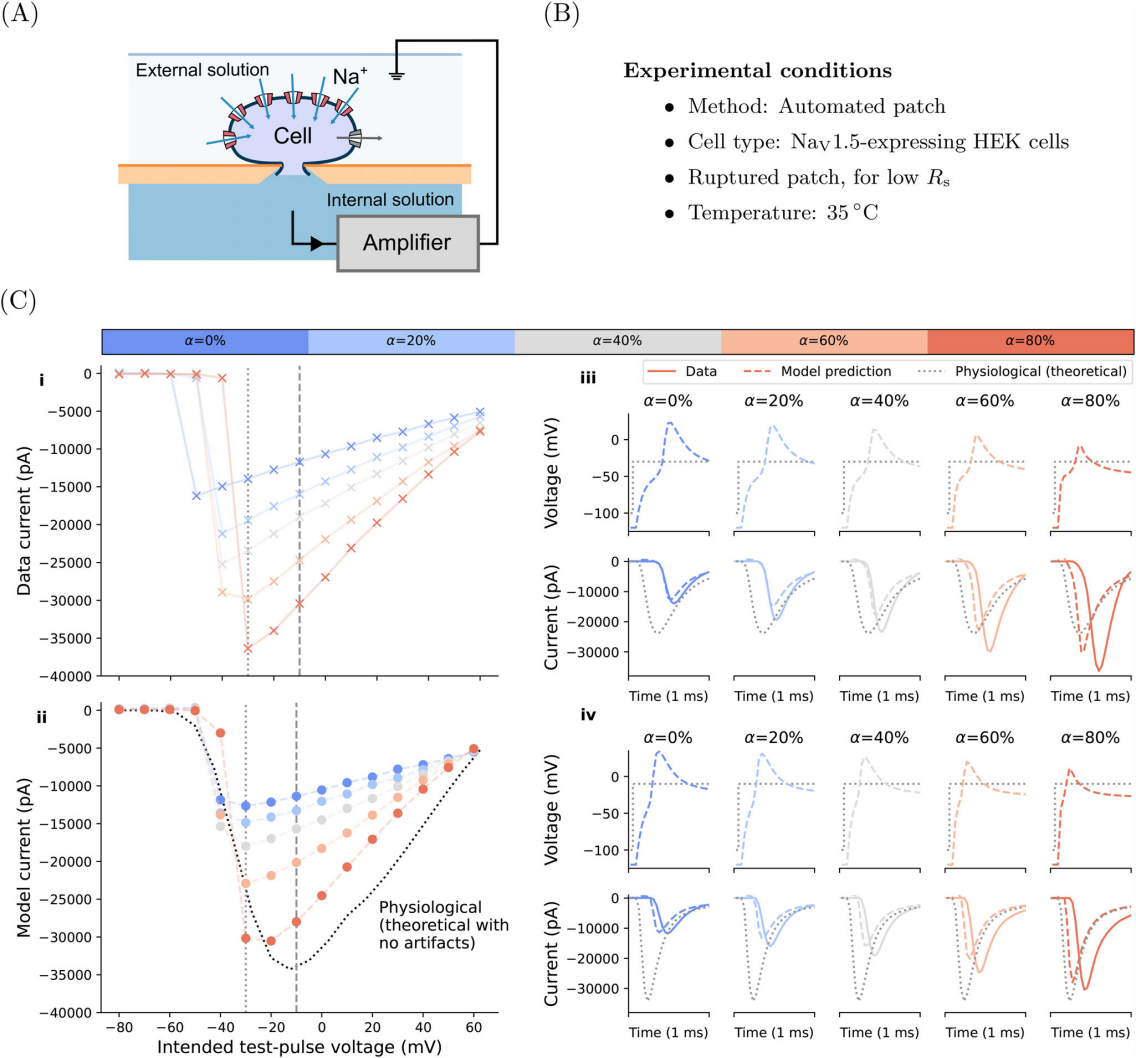
Correction of fast sodium current experiments using the computational voltage‐clamp model. A) A schematic of the experimental setup and B) the experimental conditions for the ruptured patch experiments with HEK cells over‐expressing Na_V_1.5. C) i: Experimental recordings (solid lines) of the current–voltage (*I*–*V*) relationships of fast sodium current, measured with different levels of series resistance and prediction compensation (α_*R*_ = α_*P*_ = α), with estimated *R*_s_ = 5.7 MΩ and *C*_m_ = 14.1 pF (*n* = 3, repeats are shown in Supporting Information). ii: Simulated (dashed lines) *I*–*V* relationships. iii, iv: Membrane voltage and measured and simulated current during the step to −30 mV (iii, indicated by the vertical dotted line in panels i and ii) and −40 mV (iv, indicated by the vertical dashed line in i and ii). The figure shows the effect of increasing compensation levels simultaneously (α_*R*_ = α_*P*_). Experimental recordings are shown with solid colored lines, simulated effects shown in dashed colored lines, and ideal simulated fast sodium current shown with black dotted lines. Note how the strong deviation of the voltage clamp from the ideal *V*_cmd_ (shown in (C) iii & iv) is only accessible and revealed via the simulation.

To demonstrate the approach with in vitro experimental data, we used HEK cells over‐expressing Na_V_1.5 under rupture‐patch conditions, in order to achieve low *R*_s_ in the experiments (see Experimental Section). Unlike the previous section, where strong experimental artifacts were desired to assess computational model performance, conditions were as close to physiological as practical (**Figure** **5**A, B). The *I*–*V* relationships of the fast sodium current were measured under different levels of compensation, from none to high (80 %) for both settings (α_*R*_ = α_*P*_). Compensation had a profound effect on the *I*–*V* relationship, visible in both the experimental data and the mathematical model in Figure 5. Increasing the compensation level not only elevated the maximum current but also right‐shifted the peak of the *I‐*‐*V* curves in both the experiment and the simulations. Additionally, the predicted artifact‐free *I‐*‐*V* curve (from simulations of just the ionic current with an ideal clamp) markedly deviated from the simulations of 80% compensation with the voltage‐clamp model. Our computational approach therefore provides a way to understand the underlying physiological *I‐*‐*V* relationships of Na_V_1.5, unobtainable by purely experimental means.

Figure 5C (iii‐iv) shows the current and membrane voltage of two of the voltage steps where the experimental current peaked (−30 mV) and where the artifact‐free current reconstruction peaked (−10 mV). Here, the differences caused by different compensation levels are explained by visualizing the loss of voltage clamp: the cell experienced different voltages when clamped with different compensation levels, resulting in different current dynamics. More repeats of the in vitro experiments are shown in Supporting Information.

### 2.5 Averaging *I*–*V* Curves does not Eliminate Experimental Artifacts

**Figure 6:**
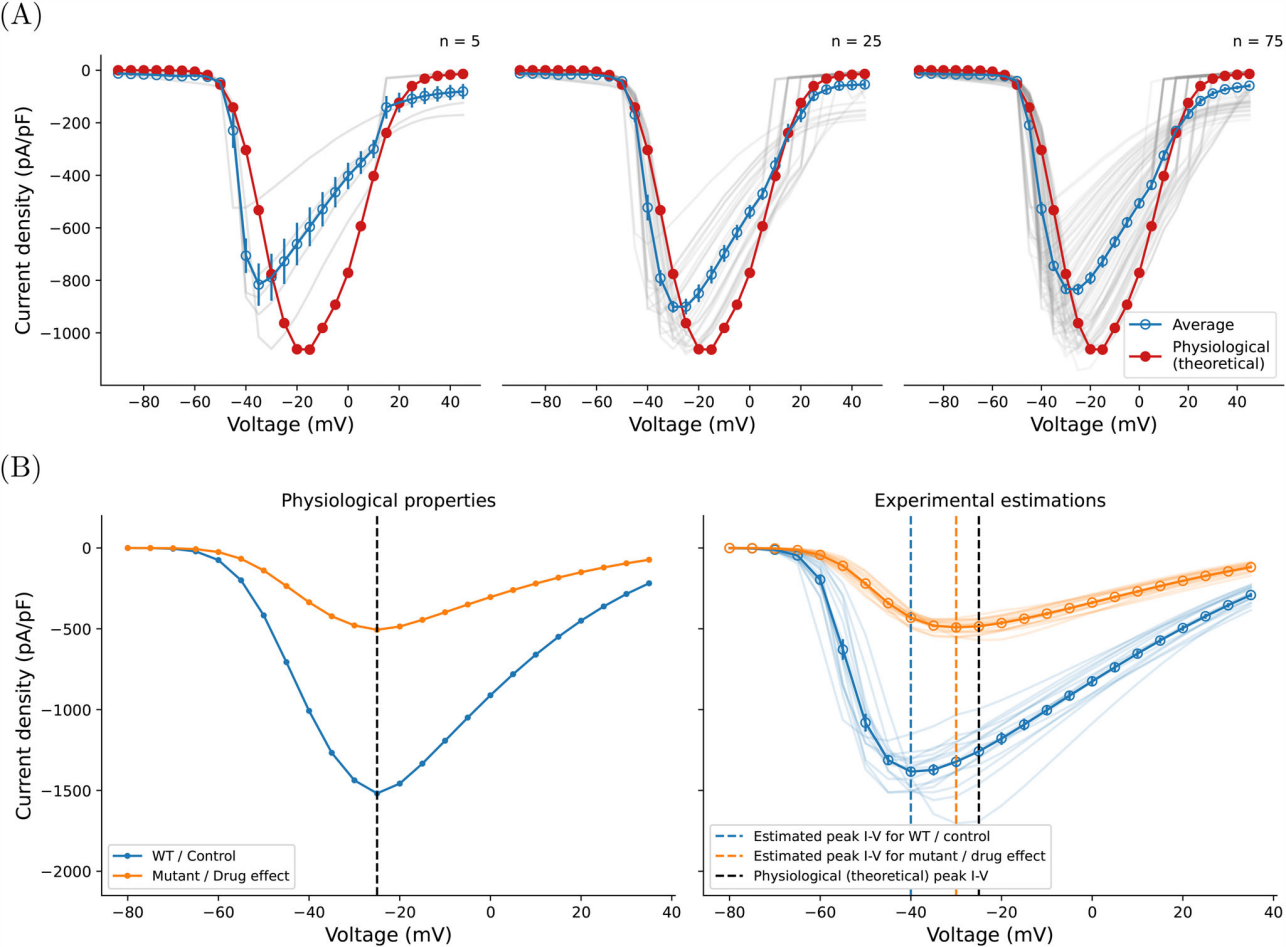
Consequences of averaging fast sodium current *I*–*V* curves of multiple runs of experiments demonstrated using simulated experiments. A) Comparing the average simulated *I*–*V* curve (blue with error bars showing SEM; averaged from transparent gray lines) to the artifact‐free physiological model *I*–*V* curve (red), neither the peak current, the peak voltage, nor the gradient of the curve of the average *I*–*V* matches the physiological *I*–*V* curve. Left to right shows different number of repeats, *n* = 5, 25, and 75, respectively. B) A simulated example of how experimental artifacts may lead to mischaracterization of mutants or drug effects. Left: The true underlying physiological *I*–*V* curves — from simulations with no artifacts — of the mutant or the drug (orange) compared to the WT or control (blue), where only current conductance was reduced by one‐third. Right: The observed *I*–*V* curves of the two conditions under one of the most stringent experimental conditions (only samples of *R*_s_ < 4 MΩ were allowed). The solid lines are the mean of the individual *I*–*V* curves (*n* = 15) shown in transparent lines, with error bars showing the SEM. Vertical dashed lines show the voltage at which *I*–*V* curves peak, with blue for WT / control, orange for mutant / drug, and black for the true effect.

Finally, we found that conventional averaging of *I*–*V* curves can obscure the problems of imperfect compensation and present a deceptive view of the underlying physiology. **Figure** **6**A shows the results of taking averages of simulated *I*–*V* curves compared to the underlying physiological model *I*–*V* curve. Each individual *I*–*V* curve was computed by using the voltage‐clamp model at α_*P*_ = α_*R*_ = 80 % and the O'Hara et al.^[^ ^23^ ^]^ fast sodium current model. The maximum conductance were scaled by values sampled from a Latin hypercube algorithm with boundaries [0.2, 5], and membrane capacitance and series resistance were set to values sampled within [8, 22] pF and [4, 15] MΩ, respectively. These settings reflect typical patch‐clamp conditions for Na_V_1.5 over‐expressing cell lines of smaller cells, such as HEK or Chinese Hamster Ovary (CHO) cells. The capacitance and series resistance were assumed to be estimated perfectly ($C_{m}=C_{m}^{*}$ and $R_{s}=R_{s}^{*}$).

We tested with a typical sample size *n* = 5, and then with increased sample counts of *n* = 25 and *n* = 75. In all cases the maximum peak current was underestimated by 10 % to 30 %, the peak voltage was left‐shifted by about 10 mV to 25 mV, and the gradient of the *I*–*V* were underestimated by 50 % to 60 %. The results show that increasing the number of samples does not resolve the issue. Simply taking means of the 80 % compensated *I*–*V* curves produces a smooth *I*–*V* curve that appears to be physiological, even though all the underlying curves show the steep gradients on the left associated with loss of voltage clamp. The resulting averaged *I*–*V* curves remain a poor approximation of the physiological relationship (Figure 6A).

Additionally, note that the experimental ‘error bar’ limits (standard errors of the mean, SEM) also do not contain the true physiological values. Even looking at the distribution of underlying *I*–*V* curves, the physiological values are outside the first standard deviation (see Supporting Information). Finally, we note that normalizing *I*–*V* curves before averaging did not resolve the issue either, as shown in Supporting Information.

**Figure 7:**
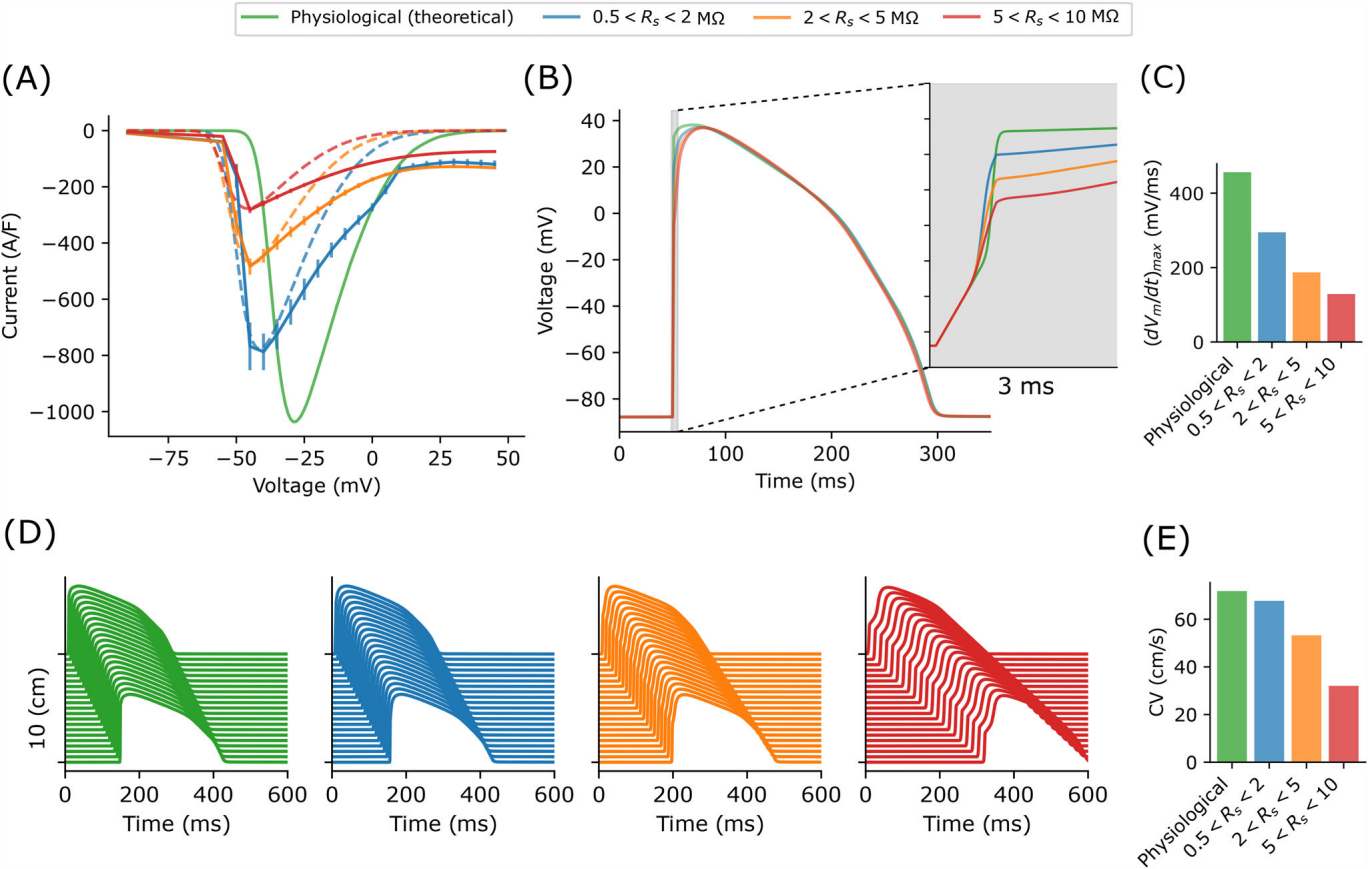
Consequences of averaging fast sodium current *I*–*V* curves of multiple runs of experiments in cell level action potential and cable simulations. A) Fitting results (dashed lines) to the average *I*–*V* curves (solid lines, error bars showing SEM with *n* = 50) simulated with different *R*_s_ conditions; blue: 0.5 < *R*_s_ < 2 MΩ, orange: 2 < *R*_s_ < 5 MΩ, and red: 5 < *R*_s_ < 10 MΩ, assuming measurements are done with cardiomyocytes with *C*_m_ between 50 pF and 150 pF. The results show how the peak current and the peak voltage were shifted away from the physiological *I*–*V* curve (green) as *R*_s_ increases, but in most literature data, *R*_s_ values were between the blue and the orange cases; even the best scenario (blue) would result in a shift of the peak *I*–*V* by −13 mV and a reduction of peak current by 30 %. B) The consequences such imperfect characterizations of the fast sodium current in an action potential model, shown with a magnification of the upstroke where the sodium current took place. C) The impacts of artifact in determining the maximum upstroke velocity (d*V*_m_/d*t*)_max_, where even the best scenario (blue) was reduced by one‐third. D) Cable simulations for the action potential models in (B), demonstrating the impacts of mischaracterization in cable conduction across 1000 cells (10 cm). E) Conduction velocity (CV) produced based on the fast sodium current characterized under different *R*_s_ conditions.

To further explore the consequences of these experimental artifact in characterizing adult human cardiomyocytes, we repeated the simulations of *I*–*V* curves using conditions representing experiments with cardiomyocytes. **Figure** **7**A shows the averaged *I*–*V* curves in solid lines with error bars showing SEM. Individual *I*–*V* relationships were simulated using the voltage‐clamp model at α_*P*_ = α_*R*_ = 80 % and the Gray and Franz^[^ ^24^ ^]^ fast sodium current model as the baseline model with the maximum conductance set to produce maximum current magnitude of ≈1000 pA/pF in the *I*–*V* curve. The maximum conductances were scaled by values sampled from a Latin hypercube algorithm with boundaries [0.2, 5], and the membrane capacitances were set to values within [50, 150] pF, emulating typical conditions of adult cardiomyocytes.^[^ ^25^ ^]^ Different series resistance conditions were tested here, with (1) 0.5 < *R*_s_ < 2 MΩ shown in blue, (2) 2 < *R*_s_ < 5 MΩ in orange, and (3) 5 < *R*_s_ < 10 MΩ in red; most of the literature data for the fast sodium current recommended using conditions between (1) and (2). The results show that the simulated conditions were shifted further away from the physiological *I*–*V* curve (green) as *R*_s_ increases, and even the best scenario (blue) produced a shift of the peak *I*–*V* by −13 mV and a reduction of the peak current by 30 %.

We fitted the Gray and Franz^[^ ^24^ ^]^ fast sodium current model without considering voltage‐clamp artifacts to the simulated averaged *I*–*V* curves, shown as dashed lines in Figure 7A, by matching the peak of the I‐V curves. The consequences in action potentials were studied by replacing the fast sodium current of the Dutta et al.^[^ ^26^ ^]^ action potential model with the fitted models based on different experimental conditions (Figure 7B). Most noticeably, the upstrokes of the action potentials were heavily affected, where the influx of sodium occurred. The maximum upstroke velocity / rate (d*V*_m_/d*t*_max_) was reduced by one‐third even with the best experimental conditions (Figure 7C). Cable simulations of these action potentials were performed to observe the effects in action potential propagation (Figure 7D). A total of 1500 cells were connected in series with gap junction conductance set to 14 nS/pF to produce a conduction velocity (CV) of $71\;{\mathrm{cms}}^{-1}$ ^[^ ^27^ ^]^ in the physiological (baseline) condition, and only the central 1000 cells (10 cm) were shown and analyzed to avoid any boundary effects. Reduced CV is associated with a heightened risk of re‐entrant excitation, which predisposes to cardiac arrhythmias.^[^ ^28^ ^]^ Figure 7E shows reductions of CVs due to imperfect characterizations of the fast sodium current under different artifact conditions, potentially more than 50 % underestimation of CV for experimental condition with 5 < *R*_s_ < 10 MΩ.

### 2.6 Experimental Artifacts can Lead to Mischaracterization of Mutants or Drug Effects

Finally, we illustrate the potential scientific impact of unaccounted‐for experimental artifacts. Consider an investigator studying a Na_V_1.5 mutant, or a pharmaceutical compound, that only reduces current conductance without affecting kinetics, e.g., a trafficking defect or simple pore block. To characterize its effect, patch‐clamp voltage‐clamp experiments are performed and compared to wild type (WT) or drug‐free controls in the same experimental settings. In our example, shown in Figure 6B, the mutant or drug block reduces the current to one‐third of its control value, as in (e.g.,) Amin et al.^[^ ^29^ ^]^

To mimic voltage clamp artifacts, we sampled experimental conditions from *R*_s_ ≈ LogNormal(2.5, 1.5) MΩ, *C*_m_ ≈ LogNormal(40, 10) pF, and $V_{\mathrm{off}}^{†}\approx \;\text{Normal}\left(0,2.5\right)$ mV, where LogNormal(μ, σ) and Normal(μ, σ) are log‐normal and normal distributions with sample mean μ and sample variance σ^2^, while each virtual cell has the same current density. The resulting simulated measurements are shown Figure 6B (right).

As the figure shows, the recorded *I*–*V* relationships (*n* = 15) are deceiving, the reduction in current amplitude causes a reduction in voltage‐clamp artifacts, but appears as a mutant or a drug that also shifts the *I*–*V* curve by 10 mV. This occurs even though we chose our voltage‐clamp model settings to match stringent experimental conditions, where only *R*_s_ < 4 MΩ pass quality control (full details in **Experimental Section**).

### 2.7 Artifacts are Sensitive to Details of Ionic Current Gating

**Figure 8:**
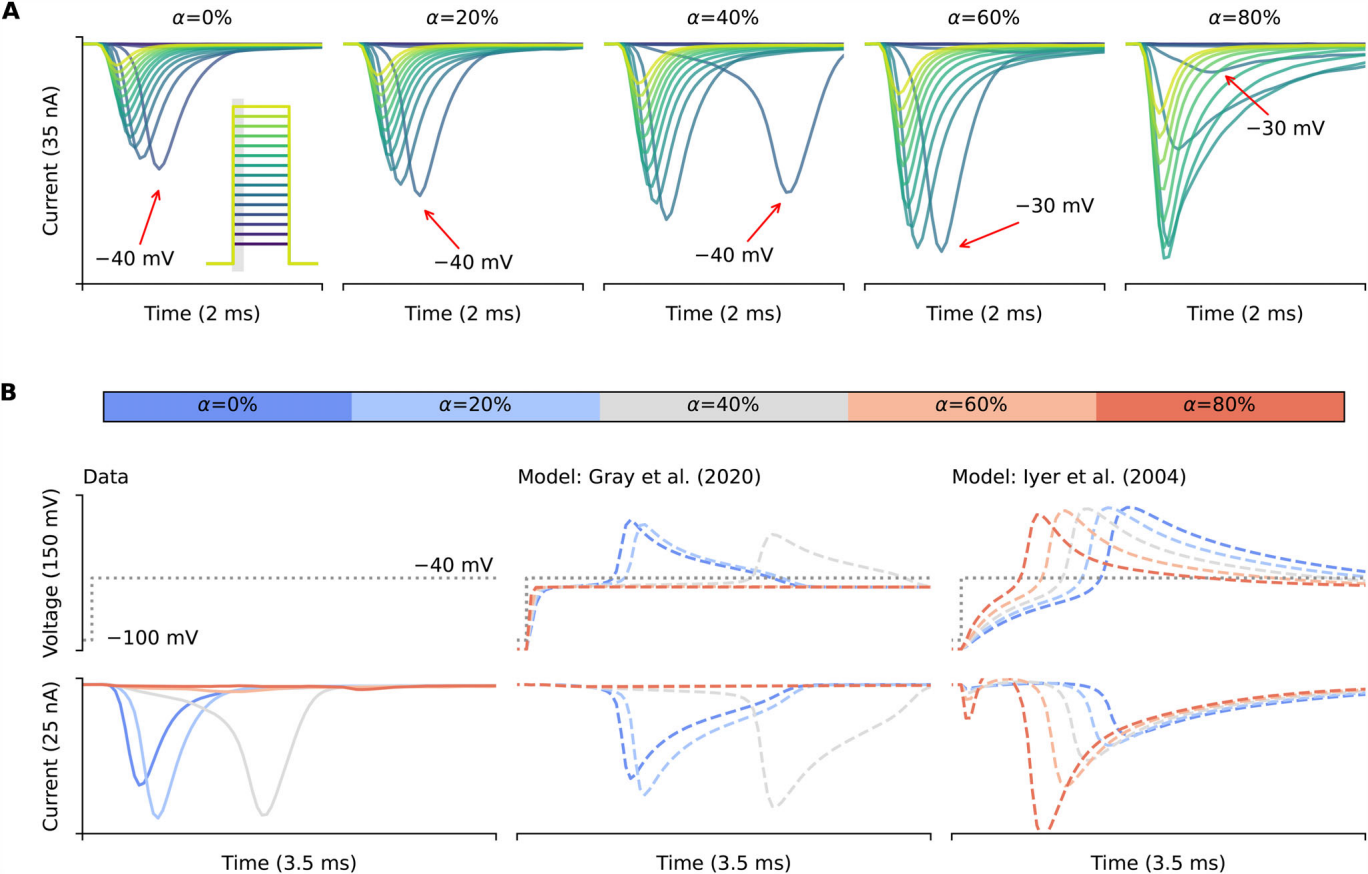
The voltage‐clamp model can qualitatively capture the delayed current of fast sodium current, but this is sensitive to the details of the gating kinetics of the ionic current model. A) Experimental recordings of fast sodium current activation at different levels of compensation with Na_V_1.5‐expressing HEK cells and the ruptured patch technique at 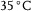, where estimated *R*_s_ = 4.3 MΩ and *C*_m_ = 10.7 pF. It shows that near the threshold voltage of current activation (≈−30 to −40 mV), there are delays in the peak time of the current as indicated by red arrows. Inset, the voltage protocol, with holding potential at −100 mV. B) An examination of the −40 mV test step. From left to right are experimental data (true membrane voltage is unknown) and voltage‐clamp model simulations with Gray and Franz^[^ ^24^ ^]^ and Iyer et al.^[^ ^22^ ^]^ fast sodium models, respectively, with different levels of compensation (α_R_ = α_P_ = α). Note the good qualitative agreement between the compensation levels that cause large loss of voltage clamp and delayed sodium activation in the Gray and Franz^[^ ^24^ ^]^ model and experimental recordings, but poor correspondence with the Iyer et al.^[^ ^22^ ^]^ model.

Our results have shown that not only the I‐V relationships but also the time‐to‐peak can be affected by artifacts (Figure 4). However, such effects on the time‐to‐peak are neither linear nor straightforward, in fact, when close to the fast sodium activation threshold voltage, the artifacts are difficult to predict and depend on the precise model used for channel kinetics. **Figure** **8**A shows the experimental recordings of the fast sodium current at different compensation levels (left to right). The red arrows indicate the recorded delays of the peak currents at around −30 to −40 mV. Depending on the conditions, the delay can be longer than the time taken for inactivation, see for example α = 40 %. Even for the typical experimental conditions, with α = 80 %, we can still observe weaker but significant delays in some of the peaks. These phenomena can be explained using our computational models, as shown in Figure 8B. Using the sodium model from Gray and Franz^[^ ^24^ ^]^ together with our voltage‐clamp mathematical model (middle panel), we are able to qualitatively reproduce the seemingly unpredictable delay patterns (compare experimental data in bottom left panel with model predictions in bottom center panel). Our model reveals that the delays were due to the nonlinear interactions between the loss‐of‐clamp of the membrane voltage and the sudden large current activated near the threshold voltage, as shown in the middle panel of Figure 8B.

A potential explanation of seemingly unpredictable time‐to‐peak delay in some fast sodium recordings was shown to be due to a positive feedback between the current and the loss of clamp in membrane voltage. Due to the nature of a very steep activation *I*–*V* relationship of the sodium current, when the voltage is set to just below the activation threshold voltage, a small negative current from the cell induces a small positive voltage due to the series resistance effect. This induction further induces a slightly bigger sodium current to push the membrane voltage even closer to the activation threshold. This positive feedback will eventually set the membrane voltage to hit the activation threshold of the sodium current, which results in the large current spike with a delay. This time‐to‐peak delay will depend on the series resistance and its compensation, as well as the slope of the sodium I‐V activation curve.

Although simulations with the Gray and Franz^[^ ^24^ ^]^ model qualitatively match experimental delays due to loss‐of‐clamp of membrane voltage, this behavior is sensitive to the choice of model. We were not able to reproduce the observed delay patterns when replacing the ionic current model with the fast sodium current from Iyer et al.^[^ ^22^ ^]^ (see Figure 8B, right). We believe this is due to model current–voltage relationships near −30 to −40 mV needing to be highly realistic to reproduce the delay patterns.

## 3 Discussion

Patch‐clamp voltage clamp is the gold standard for cellular electrophysiology. Yet pervasive experimental errors, that persist even after best‐practice compensations, can lead to artifacts in literature data. The rise of automated patch clamp means data with slightly worse seals and higher series resistance is more widely‐used than ever. Here, effects of patch artifacts were demonstrated and explained through a new computational model of voltage‐clamp experiments, validated through experiments with a newly‐designed physical hardware ‘electrical model cell’ circuit. Using fast sodium current as an example, we showed that averaging data from multiple experiments does not eliminate artifacts, but leaves a systematic bias, which can cause erroneous conclusions in settings such as characterization of mutants or drugs.

Simulations using our mathematical voltage clamp model provide a way to estimate these effects, and therefore correct for them. Previous methods to perform such corrections have relied on amplifier circuitry, which we showed were inadequate in some situations. Alternatively, to reduce current flow and the size of artifacts researchers have been forced to employ less physiological conditions — such as recording at lower temperatures, or using unphysiological solutions. However, we have previously shown how extrapolating from unphysiological to physiological temperatures can produce biased results, which cannot be corrected with simple Q_10_ scalings.^[^ ^30^ ^]^ Similar effects may occur if channel kinetics or maximal conductances are sensitive to ion concentrations as well. In common with Abrasheva et al.,^[^ ^20^ ^]^ we argue that computational models and simulations offer a new way to quantitatively use more heavily‐polluted data, enabling the study of more physiological conditions.

In our analysis of the voltage‐clamp model subject to different compensation settings in hiPSC‐CMs, we observed independent effects of series resistance compensation and prediction, confirmed by experimental data (Figure 4). Although one would not use compensation levels (α) less than 70 % when studying the physiological properties of the cells (but also typically not higher than 85 %), our results reproduce the trend of the compensation effects, providing a qualitative indication of the likely over / under estimation of recorded currents through time. The observed shifts and biases were due to imperfect clamp, or even loss of control, of the membrane voltage — we expect other derived biomarkers, such as apparent reversal potentials, would also be shifted. These effects are systematic biases (Figure 6A) which cannot be eliminated by simply averaging multiple repeats of the same experiment, which is typical practice, and can lead to incorrect estimations of cell properties.

In a previous study, we examined the role of patch clamp artifacts on recordings of a current with slower gating kinetics — cardiac I_Kr_ or hERG.^[^ ^17^ ^]^ Small rapid artifacts, such as those associated with supercharging and pipette capacitance, will have less impact on the characterization of slower currents (which is why supercharging was not included in the previous version of the patch‐clamp model ^[^ ^17^ ^]^). On the other hand, a previous study^[^ ^20^ ^]^ attempted to examine the role of supercharging in fast sodium current but did not include series resistance which resulted in nonlinear compensation effects as demonstrated in Figure 3. But noteworthy, in these situations, artifact‐induced errors can still occur — particularly when imperfect series resistance compensation causes persistent membrane voltage offsets in the presence of large persistent ionic currents. The presented patch‐clamp model allows both fast and slow artifacts to be simulated and studied.

This computational framework not only improves interpretation of new experiments, but can be used to reexamine previously published data — provided sufficient technical experimental detail is available. To accurately fit parameters that describe the true membrane voltage and ionic current dynamics, it is essential that both the raw current traces and the associated artifact‐relevant measurements / settings — including series resistance, membrane capacitance, seal resistance, and compensation settings — are reported on a per‐cell basis. Several studies have begun to adopt this level of reporting,^[^ ^31^, ^32^, ^33^ ^]^ and we believe this should become standard practice. Without these cell‐specific data, it is not possible to accurately simulate the voltage‐clamp artifacts or recover the underlying biophysical parameters.^[^ ^17^ ^]^ Sharing such detailed data will become increasingly important with the increased usage of automated patch systems which are more prone to artifacts. Broader adoption of this practice would substantially enhance the utility of archival datasets and allow systematic correction of voltage‐clamp distortions across studies.

A limitation of the approach, discussed above and shown in Figure 8, which is also shared by previous studies,^[^ ^17^, ^18^, ^20^ ^]^ is the reliance on the assumption of a known and accurate mathematical ion current gating model. Any discrepancy between the mathematical ion channel model and the real currents^[^ ^34^, ^35^ ^]^ may cause nonlinear disruption. Note also that the voltage‐clamp model captures the first‐order behavior of the amplifier components, but some of the timescales of the interactions between amplifier electrical components are in the order of microseconds or in a very high frequency domain which are not essential for correcting the experimental errors and biases for most of the systems of interest. For example, some of the amplifiers use a specific type of low‐pass filter, such as a 4‐pole low‐pass Bessel filter on the HEKA EPC 10 amplifier, in place of Equation (9). There may also be other terms such as nonlinear leak currents in some automated patch clamp,^[^ ^36^ ^]^ not included in our model, that may be setup or experiment specific. Therefore, Equation (1)–(10) form a first‐order approximation to the overall effect but this should be sufficient for capturing and correcting the majority of experimental errors.

## 4 Conclusion

In conclusion, our new modeling approach provides a step forward in how voltage‐clamp electrophysiological data can be interpreted, making fitting, and thus recovering, accurate estimates of biophysical ion current properties possible with a wider range of experimental conditions than before.

## 5 Experimental Section

### Derivation of the Mathematical Compensation Model

The derivation of part of the mathematical voltage clamp model was described in a previous publication,^[^ ^17^ ^]^ and the derivation of the full new model was provided in Supporting Information. Here, the updated equations for series resistance compensation were described that build upon Lei et al.^[^ ^17^ ^]^ by including prediction / supercharging compensation.

The series resistance *R*_s_ had two effects on the recordings: 1) it caused a persistent difference between *V*_m_ and *V*_cmd_ (i.e., exhibited even with steady currents); 2) it caused an additional transient error in *V*_m_ by reducing the speed at which it approaches its new target value after a change in *V*_cmd_.

The first, persistent, effect was proportional to the total current (*I*_ion_ + *I*_leak_), and could be reduced through series resistance compensation.^[^ ^9^, ^10^, ^15^ ^]^ This adds a compensation term to the command potential to compensate for the persistent voltage drop over *R*_*s*_, replacing *V*_cmd_ with $V_{\mathrm{cmd}}+\alpha_{R}R_{s}^{*}I_{\mathrm{out}}$, where $R_{s}^{*}$ was the machine estimation of the series resistance *R*_s_, and α_*R*_ was the user‐specified proportion of series resistance compensation.

The second, transient, effect was characterized by the product of the series resistance *R*_s_ and the membrane capacitance *C*_m_ (known as the membrane assess time constant), and can be compensated with “supercharging”,^[^ ^37^ ^]^ also known as “prediction”. In this compensation method, the target voltage was briefly given a (potentially quite large) overshoot in order to charge the membrane more rapidly (hence the name “supercharging”). The overshoot was based on an estimate (or “prediction”) of the membrane potential, *V*_est_, and was set as $\alpha R_{s}^{*}C_{m}^{*}dV_{\mathrm{est}}/dt$.

### Electrical Model Cell Validation Experiments

The electric circuit for the electrical model cell was constructed with the components listed in Figure 3A–B. The two probes were each connected to a multichannel patch‐clamp amplifier (HEKA EPC 10 USB multichannel version), one was set to voltage‐clamp mode and the other was in current‐clamp mode (with zero current injection), as shown in Figure 3A. Automated fast capacitance estimation (i.e., $C_{p}^{*}$) was applied using the HEKA PATCHMASTER software to compensate for *C*_p_, while the values of $C_{m}^{*},R_{s}^{*}$ were set to the electrical component values during the experiments. Three types of compensation settings were tested: 1) the supercharging (prediction, α_*P*_) was fixed to zero and series resistance compensation (α_*R*_) was varied from 0 % (no compensation) to 80 % with an increment of 20 %, and 95 %; 2) α_*R*_ was set to the maximum (95 %) and α_*P*_ varied from 0 % to its maximum; and 3) α_*R*_ and α_*P*_ were varied together. Experimental data were acquired with a large voltage step protocol that consists of a holding step −80 mV stepping to 50 mV for 20 ms before stepping back to the holding step. The protocol was intended to draw a large current response from the electrical model cell.

### hiPSC‐CM Perforated Patch Experiments

The perforated patch‐clamp experiments with hiPSC‐CMs (Figure 4) were from a subset of cells in Clark et al.^[^ ^31^, ^38^ ^]^ The data included in this paper (i.e., Na_V_ protocols), however, had not been previously published. The data reported here were collected after the acquisition of the current clamp and voltage clamp data in the previous publications. Here, a brief overview of the experiments was provided.

Frozen stocks of hiPSC‐CMs from a healthy individual (SCVI‐480CM) were obtained from Joseph C. Wu, MD, PhD, at the Stanford Cardiovascular Institute Biobank. The hiPSC line was derived from an African‐American female donor and was approved by Stanford University Human Subjects Research Institutional Review Board. The hiPSC‐CMs used in this study were differentiated from passage 12 hiPSCs. Amphotericin B was used as the perforating agent. Patch‐clamp measurements were made at 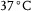 and at 10 kHz by a patch‐clamp amplifier (Model 2400; A‐M Systems, Sequim, WA) controlled by the Real Time eXperiment Interface (RTXI; http://rtxi.org) to send commands to the amplifier via the data acquisition card (PCI‐6025E; National Instruments, Austin, TX). *R*_m_, *C*_m_, and *R*_s_ values were measured at 0 mV within one minute prior to the acquisition of Na_V_ protocol data. *I*–*V* curves were acquired by stepping from −80 mV to voltages between −70 mV and 60 mV, incrementing by 10 mV. The excitation step was held for 50 ms before returning to −80 mV for 400 ms. To test multiple compensation settings, series resistance compensation (i.e., α_*R*_) was first set to 0 %, and supercharging (i.e., α_*P*_) was increased from 0 % to 80 % in 20 % increments. Then, supercharging was set to 70 %, and series resistance compensation was incrementally increased from 0 % to 80 % in 20 % increments. Compensation settings above the maximum here (α_*P*_ = 70 %, and α_*R*_ = 80 %) resulted in undesirable oscillations.

### Na_V_1.5 Experiments

Na_V_1.5‐expressing HEK‐293 cells were purchased from Charles River (CT6207). Experiments were conducted at 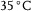 using a four‐channel Nanion Patchliner and either medium or high resistance chips. Series resistance compensation and supercharging were initially set at 0 % and incremented by 20 % up to 80 %. The *I*–*V* recordings were collected by holding at −100 mV for 1980 ms before stepping to an incrementally increasing voltage between −80 mV and 60 mV and holding for 20 ms. The internal solution included: 10 mM EGTA, 10 mM HEPES, 10 mM CsCl, 10 mM NaCl, 110 mM CsF, and with a pH adjusted to 7.2 with CsOH (>280 mOsm). The external solution included: 140 mM NaCl, 4 mM KCl, 2 mM CaCl_2_, 1 mM MgCl_2_, 5 mM D‐Glucose monohydrate, 10 mM HEPES, and with a pH adjusted to 7.4 with NaOH (298 mOsm).

### Fitting to Experimental Data

Model prediction results in Figure 5 were obtained by fitting the Iyer et al.^[^ ^22^ ^]^ fast sodium model with the voltage‐clamp model to the experimental data. Only the maximum conductance of the current and the voltage‐clamp model parameters were varied and fitted to the data, where the voltage‐clamp model parameters include *R*_s_, *C*_m_, $V_{\mathrm{off}}^{†}$, and *g*_leak_. The other parameters in the voltage‐clamp model, namely $R_{s}^{*}$, $C_{m}^{*}$, and $g_{\mathrm{leak}}^{*}$ were set to the amplifier provided estimated. Four of the fitted parameters *p*_*i*_: maximum conductance, *R*_s_, *C*_m_, and *g*_leak_ were transformed to *s*_*i*_ during the optimization using $p_{i}=p_{i}^{0}e^{s_{i}}$, where $p_{i}^{0}$ were the reference values of the parameters which were 600 nS, $R_{s}^{*}$, $C_{m}^{*}$, and $g_{\mathrm{leak}}^{*}$, respectively. The optimization was repeated 40 times at random initialization of the starting points, to ensure obtaining the global optimum solution, using the covariance matrix adaptation evolution strategy (CMA‐ES)^[^ ^39^ ^]^ accessed through the PINTS interface.^[^ ^40^ ^]^

All simulations described in this and subsequent sections were performed using Myokit 1.33.1^[^ ^41^ ^]^ with additional pre‐ and post‐processing done in Python 3.9.15 and NumPy 1.23.5.^[^ ^42^ ^]^

### Simulations of Current‐Voltage Replication

Simulations in Figure 6A were generated using the O'Hara et al.^[^ ^23^ ^]^ sodium current model with the voltage‐clamp artifact model included. *I*–*V* curves were generated by holding at −100 mV for 2000 ms before stepping to an incrementally increasing voltage between −90 and 50 mV and holding for 20 ms at each step. Both the supercharging and series resistance compensation parameters were set to 80 % for all simulations, while sodium conductance, series resistance, and capacitance was varied. Latin hypercube sampling was used to select the parameters for all models. The sodium conductances were selected from a log‐transformed scale between 0.2× and 5× the baseline values. All series resistances were between 4 MΩ and 15 MΩ and capacitances between 8 and 22 pF. The capacitance was assumed and series resistance were estimated perfectly, so $C_{m}=C_{m}^{*}$ and $R_{s}=R_{s}^{*}$.

### Action Potential and Cable Simulations

*I‐*‐*V* curves (solid lines) in Figure 7A were simulated using the Gray and Franz^[^ ^24^ ^]^ fast sodium current model (baseline maximum conductance set to 20 nS/pF) with the voltage‐clamp artifact model included. *I*–*V* curves were generated with the same protocol as previously described and averaged with *n* = 50. Both the supercharging and series resistance compensation parameters were set to 80 % for all simulations. Similar to the previous set of simulations, Latin hypercube sampling was used to select the parameters for all models. The sodium conductances were selected from a log‐transformed scale between 0.2× and 5× the baseline values, and all membrane capacitances were between 50 and 150 pF, emulating typical conditions of adult cardiomyocytes.^[^ ^25^ ^]^ Three sets of series resistance conditions were tested, values were sampled between (1) 0.5 MΩ and 2 MΩ, (2) 2 MΩ and 5 MΩ, and (3) 5 MΩ and 10 MΩ. It was assumed that the capacitance and series resistance were estimated perfectly, so $C_{m}=C_{m}^{*}$ and $R_{s}=R_{s}^{*}$.

To investigate the imperfect characterization of the fast sodium current, the Gray and Franz^[^ ^24^ ^]^ model without the voltage‐clamp artifact model was fitted to the simulated voltage‐clamp data by matching the peak of the *I*–*V* curves. The fitting was done by varying the maximum conductance of the model for matching the peak current, and by varying the model parameters *E*_*m*_ and *E*_*h*_ together for the peak voltage (dashed lines in Figure 7A). The physiological condition was the default Gray and Franz^[^ ^24^ ^]^ model with the maximum conductance value set to 20 nS/pF to produce around 1000 pA/pF in the *I‐*‐*V* curve.

Action potential simulations in Figure 7B were performed by replacing the fast sodium current in the Dutta et al.^[^ ^26^ ^]^ action potential model with the fitted Gray and Franz^[^ ^24^ ^]^ models based on different experimental conditions. The action potential models were pre‐paced for 10 s with a stimulus current of 1 ms duration and 60 pA/pF magnitude at a frequency of 2 Hz. The subsequent action potential stimulated with the same stimulus current was recorded. The maximum upstroke velocity was calculated using the second order accurate central differences of the recorded voltage trace.

Cable simulations were performed by connecting the action potential models in 1‐dimension with the gap junction current of the *i* ^th^ cell that had the membrane potential $V_{m}^{i}$ modeled with $\sum_{j}g_{\mathrm{gap}}\left(V_{m}^{i}-V_{m}^{j}\right)$, where *g*_gap_ was the gap junction conductance and $V_{m}^{j}$ was the membrane potential of the neighboring cells *j*.^[^ ^41^ ^]^ A total of 1500 cells were simulated with the gap junction conductance set to 14 nS/pF such that the cable simulation produced a conduction velocity of $71\;{\mathrm{cms}}^{-1}$ ^[^ ^27^ ^]^ in physiological (baseline) condition. The conduction velocity in the cable was calculated by assuming the length of each cell in the direction of the cable to be 100μm.^[^ ^41^ ^]^ To avoid any boundary effects, only the middle 1000 cells in the cable simulations were taken for analyses.

### Simulations of Mutations or Drug Effects

Simulations in Figure 6B were generated using the Iyer et al.^[^ ^22^ ^]^ sodium current model together with the voltage‐clamp model. The mutant or drug effect in this simulation study was assumed to have the exact same physiological properties of the WT or control condition (the original model setting) except the maximum current density was assumed to be one third of the WT / control condition. *I*–*V* curves were generated with the same protocol as previously described. The maximum current density was set to be 1500 A/F and 500 A/F for WT / control and mutant / drug effect, respectively, for the *I*–*V* curves under ideal voltage clamp. During the experimental estimation using the voltage‐clamp model, the supercharging and series resistance compensation parameters were set to 80 % for all simulations. Only *R*_s_, *C*_m_, and $V_{\mathrm{off}}^{†}$ were varied during each realization of the experiment as in the reality. To ensure the quality of the experiment realization, the parameters were sampled following *R*_s_ ≈ LogNormal(2.5, 1.5) MΩ, *C*_m_ ≈ LogNormal(40, 10) pF, and $V_{\mathrm{off}}^{†}\approx \;\mathrm{Normal}\left(0,2.5\right)$ mV for both WT / control and mutant / drug conditions, where LogNormal(μ, σ) and Normal(μ,σ) are a log‐normal distribution and a normal distribution with mean μ and variance σ^2^, respectively. The maximum conductance of the current was scaled according to the sampled *C*_m_ to give the same current density (under the ideal voltage clamp). A reasonably small 5 % estimation error was assumed for the series resistance and membrane capacitance, which was done by sampling $R_{s}^{*}\approx \mathrm{Normal}\left(1,0.05\right)\times R_{s}$ and $C_{m}^{*}\approx \mathrm{Normal}\left(1,0.05\right)\times C_{m}$.

### Statistical Analysis

Data were presented as individual cell recordings. As discussed in Figure 6, it is advised against the averaging of voltage clamp data. Furthermore, data were presented without normalization, as different current magnitudes produce different levels of experimental artifacts. Sample size (*n*) for each experiment was indicated in the corresponding figure caption.

## Conflict of Interest

The authors declare no conflict of interest.

## Supporting information

Supporting Information

## Data Availability

The data that support the findings of this study are openly available in Figshare at https://doi.org/10.6084/m9.figshare.27193878.v1, reference number 27193878. All codes are available at https://github.com/CardiacModelling/nav‐artefact‐model with a permanently archived version available in Zenodo at https://doi.org/10.5281/zenodo.15200038.

## References

[1] A. L. Hodgkin , A. F. Huxley , Nature 1939, 144, 710.

[2] A. L. Hodgkin , A. F. Huxley , J. Physiol. 1952, 117, 500. 12991237 10.1113/jphysiol.1952.sp004764 PMC1392413

[3] D. Levenstein , V. A. Alvarez , A. Amarasingham , H. Azab , Z. S. Chen , R. C. Gerkin , A. Hasenstaub , R. Iyer , R. B. Jolivet , S. Marzen , J. D. Monaco , A. A. Prinz , S. Quraishi , F. Santamaria , S. Shivkumar , M. F. Singh , R. Traub , F. Nadim , H. G. Rotstein , A. D. Redish , J. Neurosci. 2023, 43, 1074. 36796842 10.1523/JNEUROSCI.1179-22.2022 PMC9962842

[4] D. Noble , A. Garny , P. J. Noble , J. Physiol. 2012, 590, 2613. 22473779 10.1113/jphysiol.2011.224238 PMC3424720

[5] S. Langthaler , T. Rienmüller , S. Scheruebel , B. Pelzmann , N. Shrestha , K. Zorn‐Pauly , W. Schreibmayer , A. Koff , C. Baumgartner , PLoS Comput. Biol. 2021, 17, e1009091. 34157016 10.1371/journal.pcbi.1009091 PMC8219159

[6] G. R. Mirams , Drug Discovery, Evaluation: Safety, Pharmacokinetic Assays, Springer, Berlin, Germany 2023, pp. 1–33.

[7] B. Sakmann , E. Neher , Annu. Rev. Physiol. 1984, 46, 455. 6143532 10.1146/annurev.ph.46.030184.002323

[8] A. Marty , E. Neher , Single‐Channel Recording, (Eds: B. Sakmann , E. Neher ), Springer US, Boston, MA, USA 1983, pp. 107–122.

[9] F. Sigworth , J. Neurosci. Methods 1995a, 56, 195. 7538620

[10] F. Sigworth , H. Affolter , E. Neher , J. Neurosci. Methods 1995, 56, 203. 7538621 10.1016/0165-0270(94)00129-5

[11] C. Armstrong , R. Chow , Biophys. J. 1987, 52, 133. 2440491 10.1016/S0006-3495(87)83198-3 PMC1329993

[12] J. W. Moore , M. Hines , E. M. Harris , Biophys. J. 1984, 46, 507. 6498268 10.1016/S0006-3495(84)84048-5 PMC1435019

[13] A. Strickholm , J. Neurosci. Methods 1995, 61, 53. 8618426 10.1016/0165-0270(95)00021-l

[14] A. J. Sherman , A. Shrier , E. Cooper , Biophys. J. 1999, 77, 2590. 10545359 10.1016/S0006-3495(99)77093-1 PMC1300533

[15] P. Weerakoon , E. Culurciello , K. G. Klemic , F. J. Sigworth , IEEE Trans. Biomed. Circuits Syst. 2009, 3, 117. 23853203 10.1109/TBCAS.2008.2005419

[16] S. F. Traynelis , J. Neurosci. Methods 1998, 86, 25. 9894783 10.1016/s0165-0270(98)00140-x

[17] C. L. Lei , M. Clerx , D. G. Whittaker , D. J. Gavaghan , T. P. De Boer , G. R. Mirams , Philos. Trans. R. Soc., A 2020a, 378, 20190348. 10.1098/rsta.2019.0348 PMC7287334 32448060

[18] J. Montnach , M. Lorenzini , A. Lesage , I. Simon , S. Nicolas , E. Moreau , C. Marionneau , I. Baró , M. De Waard , G. Loussouarn , Sci. Rep. 2021, 11, 1. 33558601 10.1038/s41598-021-82077-8 PMC7870888

[19] C. L. Lei , PhD thesis, University of Oxford, Oxford, England 2020.

[20] V. O. Abrasheva , S. G. Kovalenko , M. Slotvitsky , S. A. Romanova , A. A. Aitova , S. Frolova , V. Tsvelaya , R. A. Syunyaev , J. Physiol. 2024, 602, 633. 38345560 10.1113/JP285162

[21] M. Paci , E. Passini , A. Klimas , S. Severi , J. Hyttinen , B. Rodriguez , E. Entcheva , Biophys. J. 2020, 118, 2596. 32298635 10.1016/j.bpj.2020.03.018 PMC7231889

[22] V. Iyer , R. Mazhari , R. L. Winslow , Biophys. J. 2004, 87, 1507. 15345532 10.1529/biophysj.104.043299 PMC1304558

[23] T. O'Hara , L. Virág , A. Varró , Y. Rudy , PLoS Comput. Biol. 2011, 7, e1002061. 21637795 10.1371/journal.pcbi.1002061 PMC3102752

[24] R. A. Gray , M. R. Franz , Am. J. Physiol.: Heart Circ. Physiol. 2020, 318, H534. 31951472 10.1152/ajpheart.00557.2019

[25] F. Mazhar , C. Bartolucci , F. Regazzoni , M. Paci , L. Dedè , A. Quarteroni , C. Corsi , S. Severi , J. Physiol. 2024, 602, 4543. 37641426 10.1113/JP283974

[26] S. Dutta , A. Mincholé , T. A. Quinn , B. Rodriguez , Progr. Biophys. Mol. Biol. 2017, 129, 40. 10.1016/j.pbiomolbio.2017.02.007 28223156

[27] E. J. Vigmond , S. Massé , C. H. Roney , J. D. Bayer , K. Nanthakumar , JACC: Clin. Electrophysiol 2025, 11, 694. 39818672 10.1016/j.jacep.2024.11.004

[28] J. H. King , C. L.‐H. Huang , J. A. Fraser , Front. Physiol. 2013, 4, 154. 23825462 10.3389/fphys.2013.00154 PMC3695374

[29] A. Amin , A. Verkerk , Z. Bhuiyan , A. Wilde , H. Tan , Acta Physiol. Scand. 2005, 185, 291. 16266370 10.1111/j.1365-201X.2005.01496.x

[30] C. L. Lei , M. Clerx , K. A. Beattie , D. Melgari , J. C. Hancox , D. J. Gavaghan , L. Polonchuk , K. Wang , G. R. Mirams , Biophys. J. 2019, 117, 2455. 31451180 10.1016/j.bpj.2019.07.030 PMC6990152

[31] A. P. Clark , S. Wei , D. Kalola , T. Krogh‐Madsen , D. J. Christini , Br. J. Pharmacol. 2022, 179, 4829. 35781252 10.1111/bph.15915 PMC9489646

[32] A. P. Clark , S. Wei , D. J. Christini , T. Krogh‐Madsen , J. Physiol. 2024, 602, 5163. 38747042 10.1113/JP285120 PMC11493530

[33] D. V. Van de Sande , I. Kopljar , A. Maaike , A. Teisman , D. J. Gallacher , L. Bart , D. J. Snyders , L. Leybaert , H. R. Lu , A. J. Labro , Channels 2021, 15, 239. 33465001 10.1080/19336950.2021.1871815 PMC7817136

[34] J. Brynjarsdóttir , A. O'Hagan , Inverse Probl. 2014, 30, 114007.

[35] C. L. Lei , S. Ghosh , D. G. Whittaker , Y. Aboelkassem , K. A. Beattie , C. D. Cantwell , T. Delhaas , C. Houston , G. M. Novaes , A. V. Panfilov , P. Pathmanathan , M. Riabiz , R. W. dos Santos , J. Walmsley , K. Worden , G. R. Mirams , R. D. Wilkinson , Philos. Trans. R. Soc., A 2020, 378, 20190349. 10.1098/rsta.2019.0349 PMC7287333 32448065

[36] C. L. Lei , A. Fabbri , D. G. Whittaker , M. Clerx , M. J. Windley , A. P. Hill , G. R. Mirams , T. P. de Boer , Wellcome Open Res. 2020c, 5, 152. 34805549 10.12688/wellcomeopenres.15968.1 PMC8591515

[37] F. J. Sigworth , Single‐Channel Recording, 2nd ed., (Eds: B. Sakmann , E. Neher ), Springer, Boston, MA, USA, 1995, pp. 95–127.

[38] A. P. Clark , M. Clerx , S. Wei , C. L. Lei , T. P. de Boer , G. R. Mirams , D. J. Christini , T. Krogh‐Madsen , Europace 2023, 25, euad243. 37552789 10.1093/europace/euad243 PMC10445319

[39] N. Hansen , Towards a New Evolutionary Computation: Advances in the Estimation of Distribution Algorithms, (Eds: J. A. Lozano , P. Larrañaga , I. Inza , E. Bengoetxea ), Springer, Berlin, Germany 2006, pp. 75–102.

[40] M. Clerx , M. Robinson , B. Lambert , C. L. Lei , S. Ghosh , G. R. Mirams , D. J. Gavaghan , J. Open Res. Software 2019, 7, 23.

[41] M. Clerx , P. Collins , E. de Lange , P. G. A. Volders , Progr. Biophys. Mol. Bio. 2016, 120, 100. 10.1016/j.pbiomolbio.2015.12.008 26721671

[42] C. R. Harris , K. J. Millman , S. J. van der Walt , R. Gommers , P. Virtanen , D. Cournapeau , E. Wieser , J. Taylor , S. Berg , N. J. Smith , R. Kern , M. Picus , S. Hoyer , M. H. van Kerkwijk , M. Brett , A. Haldane , J. F. del Río , M. Wiebe , P. Peterson , p. Gérard‐Marchant , K. Sheppard , T. Reddy , W. Weckesser , H. Abbasi , C. Gohlke , T. E. Oliphant , Nature 2020, 585, 357. 32939066 10.1038/s41586-020-2649-2 PMC7759461

