# Supplementary material for: Resolving Artifacts in Voltage‐Clamp Experiments with Computational Modeling: An Application to Fast Sodium Current Recordings
Source: Adv Sci (Weinh). 2025 Jun 6;12(30):e00691. doi: 10.1002/advs.202500691 (PMC12376535; doi:10.1002/advs.202500691)
Supplement: Supplementary file 1 — Supporting Information [file ADVS-12-e00691-s001.pdf]

ADVANCED SCIENCE
Open Access

Supporting Information

for *Adv. Sci.*, DOI 10.1002/advs.202500691

Resolving Artifacts in Voltage-Clamp Experiments with Computational Modeling: An Application to Fast Sodium Current Recordings

Chon Lok Lei, Alexander P. Clark, Michael Clerx, Siyu Wei, Meye Bloothooft, Teun P. de Boer, David J. Christini, Trine Krogh-Madsen and Gary R. Mirams**

Supplementary Material

Resolving artifacts in voltage-clamp experiments with computational modeling: an application to fast sodium current recordings

Chon Lok Lei, Alexander P. Clark, Michael Clerx, Siyu Wei, Meye Bloothooft, Teun P. de Boer, David J. Christini, Trine Krogh-Madsen, and Gary R. Mirams

S1 Model derivation

The derivation of the equations governing a voltage-clamp experiment *without* any compensation follows exactly as Lei et al. (2020), which is not repeated here. These include the equations for the effects of membrane capacitance, series resistance, leak current (seal resistance), pipette capacitance, and amplifier delays. Below shows the derivation of the mathematical model of how modern patch amplifiers typically compensate for them (Axon Instruments Inc., 1999; HEKA Elektronik GmbH, 2018), following the derivation from (Lei, 2020). Firstly, the voltage offset is usually estimated and compensated *prior* to adding the cell to the system, either with an automated correction estimated using *software control* or by applying manually a voltage offset such that it gives zero current when clamped at zero voltage, so the compensation circuit is not shown in our patch clamp equivalent circuits (Neher, 1995; Sigworth et al., 1995). The major source of voltage offset may be the liquid junction potential, a potential difference of $\sim$ 2–12 mV which develops when the pipette-filling solution is different from the bath solution (Neher, 1992). The adjustment is usually done by adding the theoretically estimated liquid junction potential offset to V^*_{off}. We can write the error in the estimate of the overall voltage offset $V^\dagger_{\text{off}}$ as

$$V^\dagger_{\text{off}} = V_{\text{off}} - V^*_{\text{off}}. \tag{S1.1}$$

We then simply need to replace all instances of V_{off} in the equations above with $V^\dagger_{\text{off}}$ to describe the effect of imperfect voltage offset compensation, and $V^\dagger_{\text{off}}$ is assumed to be $\mathcal{O}(10)$ mV.

Secondly, to compensate the effect of the parasitic capacitance at the electrode, an additional current is injected at the electrode to compensate for the current drawn by the parasitic capacitance. By analyzing the fast capacitance compensation part, we obtain the compensation current as $C^*_{\text{p}}\frac{\mathrm{d}V_{\text{clamp}}}{\mathrm{d}t}$ where C^*_{p} is the amplifier's estimate of the parasitic capacitance C_{p}. Then we have

$$I_{\text{m}} = I_{\text{in}} + C^*_{\text{p}}\frac{\mathrm{d}V_{\text{clamp}}}{\mathrm{d}t} - I_{\text{p}} - I_{\text{leak}} - I_{\text{ion}}, \tag{S1.2}$$

and

$$I_{\text{in}} = I_{\text{ion}} + I_{\text{leak}} + C_{\text{m}}\frac{\mathrm{d}V_{\text{m}}}{\mathrm{d}t} + \left(C_{\text{p}}\frac{\mathrm{d}V_{\text{p}}}{\mathrm{d}t} - C^*_{\text{p}}\frac{\mathrm{d}V_{\text{clamp}}}{\mathrm{d}t}\right). \tag{S1.3}$$

This is usually known as 'C-Fast' compensation.

Thirdly, we need to consider compensation for the cell membrane capacitance C_{m}. Usually the effect of C_{m} is reduced by a hardware 'C-Slow' compensation, using a similar circuit to the 'C-Fast' compensation discussed above (Sigworth et al., 1995; Sigworth, 1995a). However, since the value of C_{m} can reach 100 pF in some cell types, and capacitor sizes can be limited, the 'C-Slow' compensation is sometimes performed as a post-processing step by the amplifier control software rather than using built-in amplifier hardware (Weerakoon et al., 2010). In either case, the full capacitance compensation can be written as

$$I_{\text{in}} = I_{\text{ion}} + I_{\text{leak}} + \left(C_{\text{m}}\frac{\mathrm{d}V_{\text{m}}}{\mathrm{d}t} - C^*_{\text{m}}\frac{\mathrm{d}V_{\text{est}}}{\mathrm{d}t}\right) + \left(C_{\text{p}}\frac{\mathrm{d}V_{\text{p}}}{\mathrm{d}t} - C^*_{\text{p}}\frac{\mathrm{d}V_{\text{clamp}}}{\mathrm{d}t}\right), \tag{S1.4}$$

where C^*_{m} is the amplifier (or user's) estimate of the membrane capacitance C_{m}, and V_{est} is given below.

Finally, in voltage clamp, we want V_{m} to approach V_{cmd} as quickly as possible. However, there are two effects introduced by R_{s}, the first one causes V_{m} to deviate from V_{cmd} and the second slows down V_{m}'s approach to V_{cmd}. The first effect is caused by $(I_{\mathrm{ion}} + I_{\mathrm{leak}})$, which can be reduced through a series resistance compensation (Sigworth et al., 1995; Sigworth, 1995a; Weerakoon et al., 2009). By analyzing the series resistance compensation part, instead of clamping to V_{cmd}, it is set to $V_{\mathrm{cmd}} + \alpha R_{\mathrm{s}}^{*} I_{\mathrm{out}}$, where R_{s}^{*} is the machine estimation of the series resistance R_{s}, and α is the requested proportion of series resistance compensation (a machine setting, typically 70–85 %).

The second effect is caused by the product of the series resistance R_{s} and the membrane capacitance C_{m}, i.e. the membrane assess time constant τ_{a}, which can be reduced through a compensation termed "supercharging" (Sigworth, 1995b). We set the clamping voltage to have a large overshoot (hence the name "supercharging") proportional to $\alpha R_{\mathrm{s}}^{*} C_{\mathrm{m}}^{*}\, \mathrm{d}V_{\mathrm{est}}/\mathrm{d}t$, where

$$\frac{\mathrm{d}V_{\mathrm{est}}}{\mathrm{d}t} = \frac{1}{R_{\mathrm{s}}^{*} C_{\mathrm{m}}^{*}} \left(V_{\mathrm{cmd}} + \alpha R_{\mathrm{s}}^{*} C_{\mathrm{m}}^{*} \frac{\mathrm{d}V_{\mathrm{est}}}{\mathrm{d}t} - V_{\mathrm{est}} \right), \tag{S1.5}$$

$$= \frac{V_{\mathrm{cmd}} - V_{\mathrm{est}}}{(1-\alpha) R_{\mathrm{s}}^{*} C_{\mathrm{m}}^{*}}, \tag{S1.6}$$

according to Sigworth (1995b, Figure 18). The effect of the overshooting is to charge the membrane capacitance quickly. Including all the compensations, V_{clamp} becomes

$$\frac{\mathrm{d}V_{\mathrm{clamp}}}{\mathrm{d}t} = \frac{1}{\tau_{\mathrm{sum}}} \left(\left(V_{\mathrm{cmd}} + \alpha R_{\mathrm{s}}^{*} \left(I_{\mathrm{out}} + C_{\mathrm{m}}^{*} \frac{\mathrm{d}V_{\mathrm{est}}}{\mathrm{d}t} \right) \right) - V_{\mathrm{clamp}} \right), \tag{S1.7}$$

to counterbalance the two effects caused by the series resistance. Note that the supercharging correction is particularly important when measuring big, very fast currents such as I_{Na} which has a time-to-peak within $\sim$5 ms, however this correction poses almost no issue when analyzing smaller, slower currents, for example I_{Kr}.

S1.1 Ionic Current for Electrical Model Cell

Below, we derive the equations of the 'ionic current' (I_{ion}) for the electrical model cell circuit shown in Fig. 3A of the main text.

By analyzing the electrical components with Kirchhoff's circuit laws (Horowitz et al., 2015), we obtain

$$I_{\mathrm{ion}} = I_{\mathrm{k}} + I_{\mathrm{m}}, \tag{S1.8}$$

$$\frac{\mathrm{d}I_{\mathrm{k}}}{\mathrm{d}t} = \frac{1}{R_{\mathrm{k}} C_{\mathrm{k}}} \left(C_{\mathrm{k}} \frac{\mathrm{d}V_{\mathrm{m}}}{\mathrm{d}t} - I_{\mathrm{k}} \right), \tag{S1.9}$$

$$I_{\mathrm{m}} = V_{\mathrm{m}} / R_{\mathrm{m}}, \tag{S1.10}$$

where the values of the model cell components are given in Fig. 3B of the main text. The time constant $R_{\mathrm{k}} C_{\mathrm{k}} = 1$ ms was chosen to provide a good time scale for testing our new voltage clamp model, such as the effects of the supercharging (prediction) component. To mimic the effect of large currents such as the fast sodium current, the overall current magnitude of I_{ion} of the model cell is set to be $\mathcal{O}(10)$ nA for command voltages of $\mathcal{O}(100)$ mV.

Supplementary Tables

Symbol	Typical range	Description
General		
t	—	Time variable.
Capacitance		
C_f	0.02–1 pF	Feedback capacitance (only appears as $\tau_\mathrm{z} = R_\mathrm{f} C_\mathrm{f}$).
C_m	5–80 pF	Membrane capacitance. Typical values are given for Chinese hamster ovary (CHO) cells, HEK cells, and hiPSC-CMs.
C_p	1–10 pF	Parasitic capacitance at the pipette/well plate (and connected equipment); also known as 'pipette capacitance' in manual patch.
Current		
I_in	—	Voltage-clamp current.
I_ion	$\mathcal{O}(1)$ nA	Whole-cell ion channel current. Typical values are given for over-expressing CHO or HEK cells and hiPSC-CMs.
I_leak	—	Leakage current through imperfect seal.
I_m	—	Membrane current.
I_out	—	Recorded current.
I_p	—	Current drawn by parasitic capacitance of the pipette/well plate.
I_post	—	Post-processed (typically leak subtracted) current.
Resistance		
R_f	5 MΩ–50 GΩ	Feedback resistance (only appears as $\tau_\mathrm{z} = R_\mathrm{f} C_\mathrm{f}$).
R_s	1–30 MΩ	Series resistance between the pipette electrode and the cell.
R_seal	0.5–10 GΩ	Seal resistance of the pipette tip, $R_\mathrm{seal} = 1/g_\mathrm{leak}$.
Time constant		
τ_sum	$\mathcal{O}(1)$ µs	Response time of the summing amplifier.
τ_clamp	$\mathcal{O}(1)$ µs	Voltage-clamp time constant.
τ_z	0.1–50 µs	Transconductor time constant, $\tau_\mathrm{z} = R_\mathrm{f} C_\mathrm{f}$, set by the amplifier filtering setting, depending on the sampling frequency.
Voltage		
V_cmd	$\mathcal{O}(100)$ mV	Command voltage; follows the voltage-clamp protocols.
V_clamp	$\mathcal{O}(100)$ mV	Clamp voltage.
V_est	$\mathcal{O}(100)$ mV	Estimated (predicted) membrane potential.
V_m	$\mathcal{O}(100)$ mV	Membrane potential.
V_off	$\mathcal{O}(10)$ mV	Lumped offset voltage, such as amplifier offsets, electrode offsets, junction potentials, etc.
V_out	$\mathcal{O}(100)$ mV	Recorded voltage, $V_\mathrm{out} = I_\mathrm{out} R_\mathrm{f}$.
V_p	$\mathcal{O}(100)$ mV	Pipette potential.
Others		
α_R	0–1	Series resistance compensation level.
α_P	0–1	Supercharging compensation (or prediction) level.

Table S1: Glossary of symbols and parameters. The values are taken from Neher (1995); HEKA Elektronik GmbH (2018); Axon Instruments Inc. (1999), unless otherwise specified.

Supplementary Figures

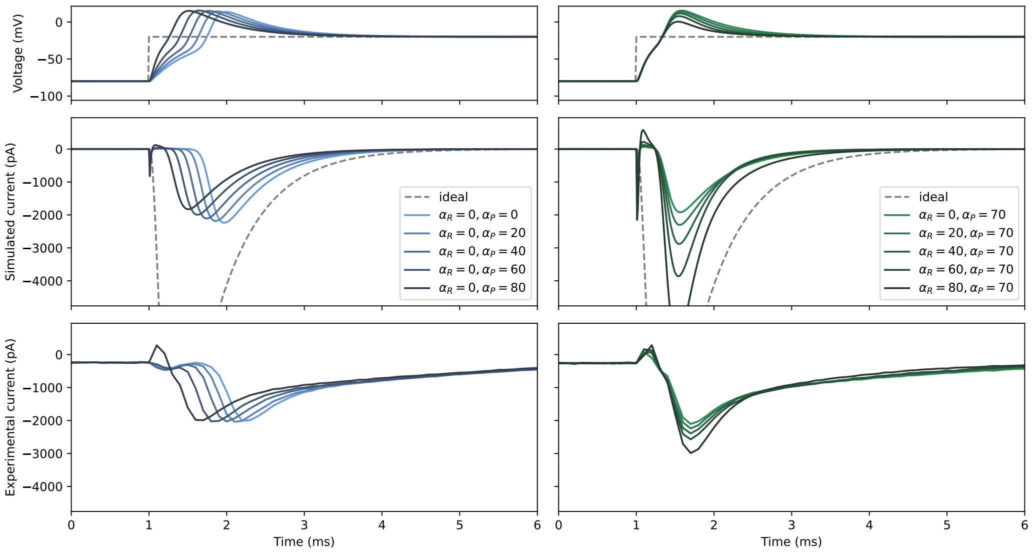

Figure S1: Forward sensitivity analysis of the experimental artifact of fast sodium current using Gray and Franz (2020) and hiPSC-CMs, with estimated $R_s = 19.5\,\mathrm{M\Omega}$ and $C_m = 27.4\,\mathrm{pF}$ (cell 1; same cell as in the main text figure).

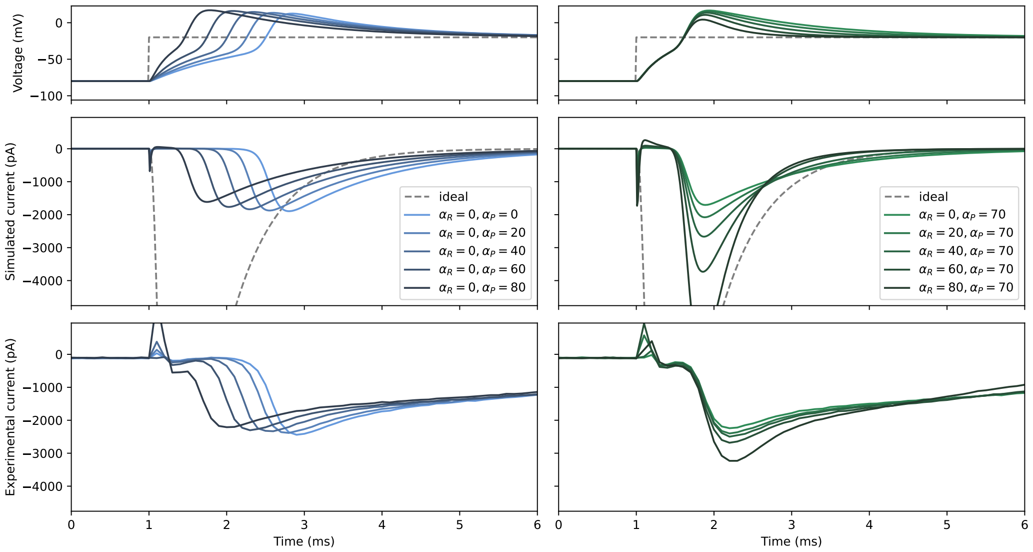

Figure S2: Forward sensitivity analysis of the experimental artifact of fast sodium current using Gray and Franz (2020) and hiPSC-CMs, with estimated $R_s = 25\,\mathrm{M\Omega}$ and $C_m = 51.5\,\mathrm{pF}$ (cell 2).

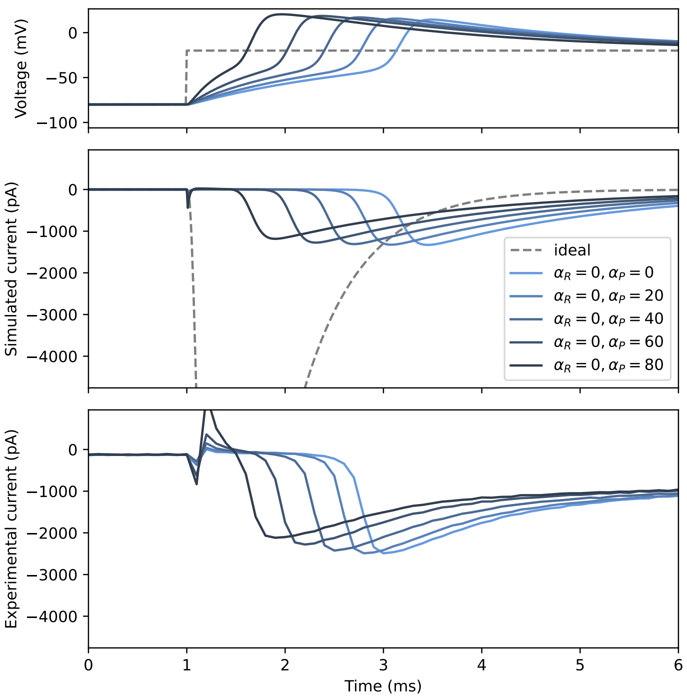

Figure S3: Forward sensitivity analysis of the experimental artifact of fast sodium current using Gray and Franz (2020) and hiPSC-CMs, with estimated $R_\mathrm{s} = 39.3\,\mathrm{M\Omega}$ and $C_\mathrm{m} = 51\,\mathrm{pF}$ (cell 3).

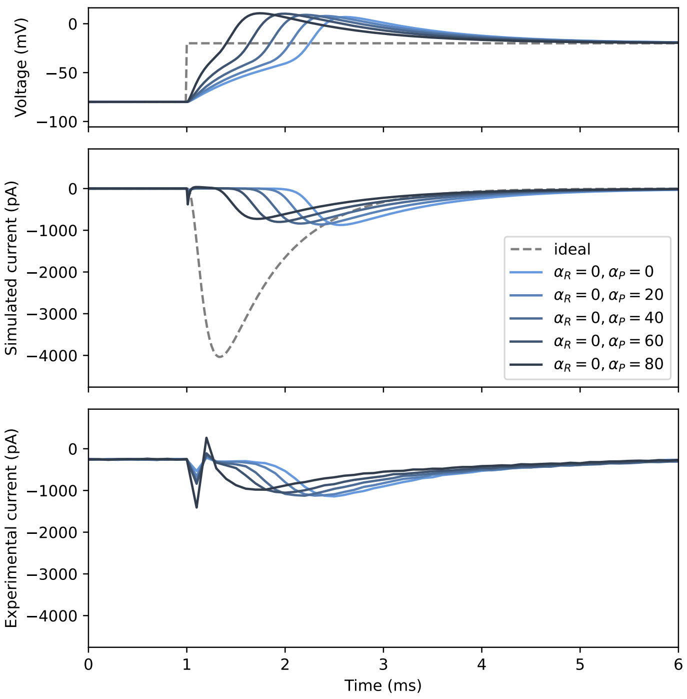

Figure S4: Forward sensitivity analysis of the experimental artifact of fast sodium current using Gray and Franz (2020) and hiPSC-CMs, with estimated $R_\mathrm{s} = 44.2\,\mathrm{M\Omega}$ and $C_\mathrm{m} = 21.7\,\mathrm{pF}$ (cell 4).

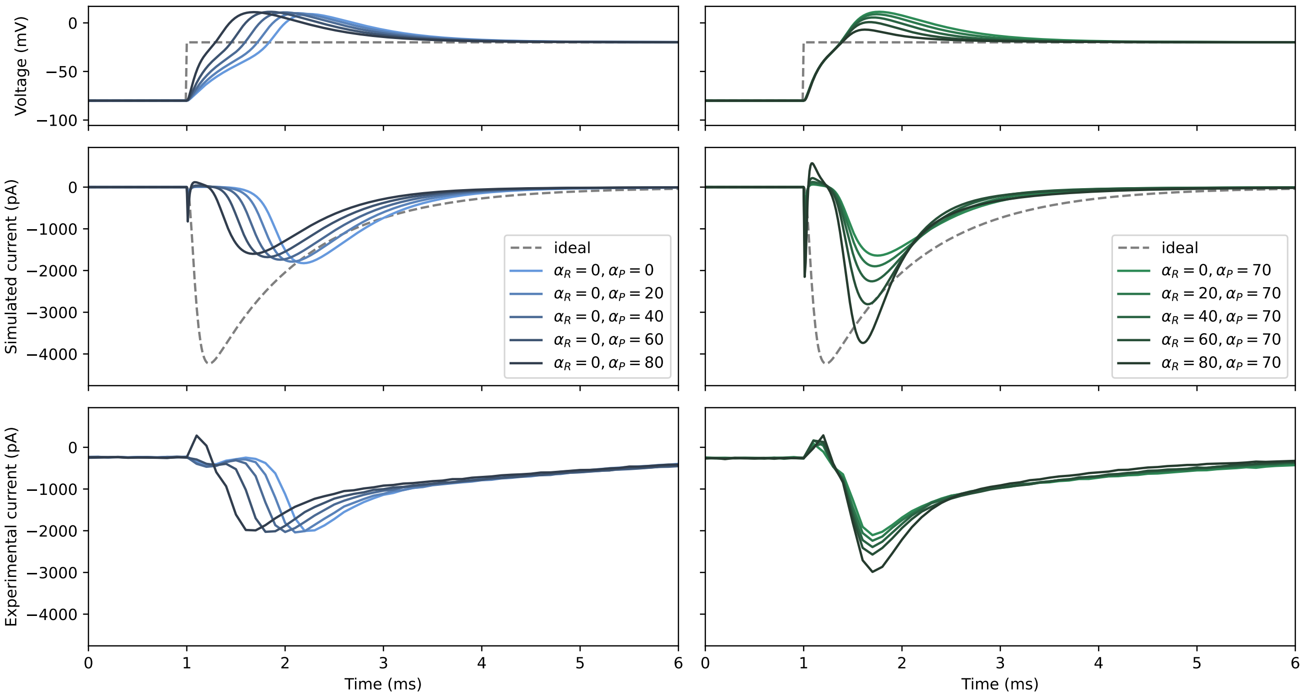

Figure S5: Forward sensitivity analysis of the experimental artifact of fast sodium current using Paci et al. (2020) and hiPSC-CMs (cell 1; same cell as in the main text figure).

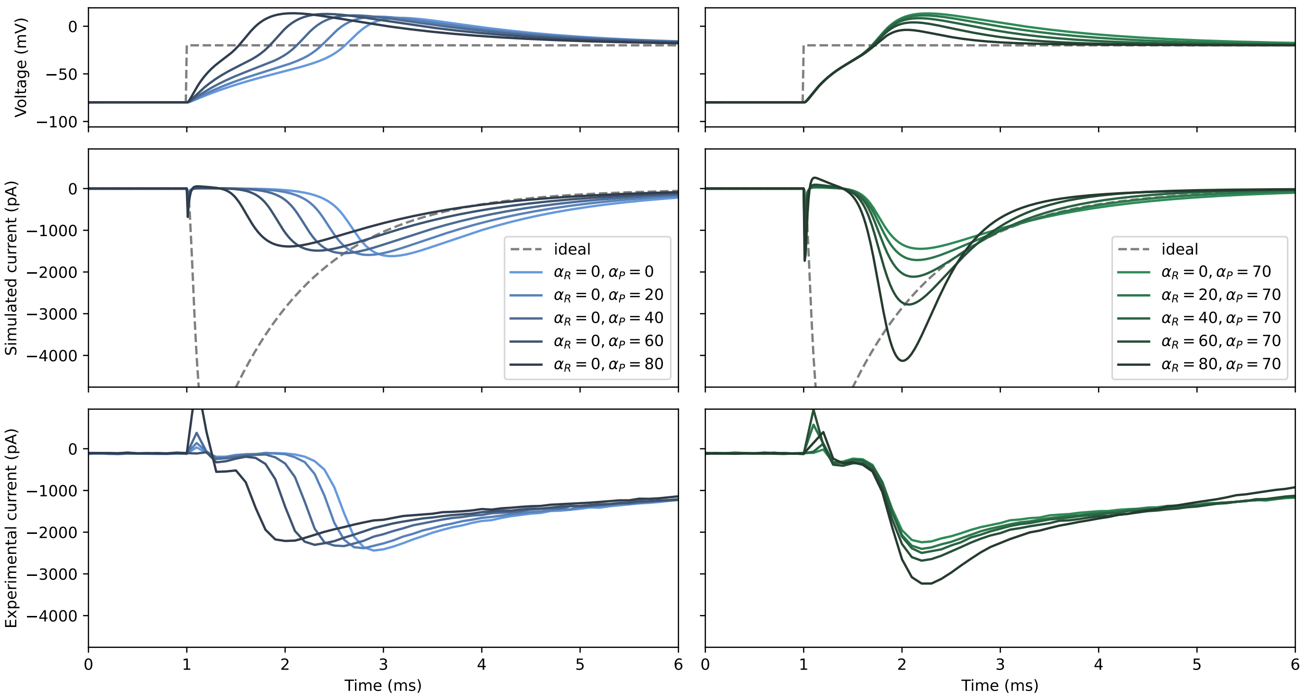

Figure S6: Forward sensitivity analysis of the experimental artifact of fast sodium current using Paci et al. (2020) and hiPSC-CMs (cell 2).

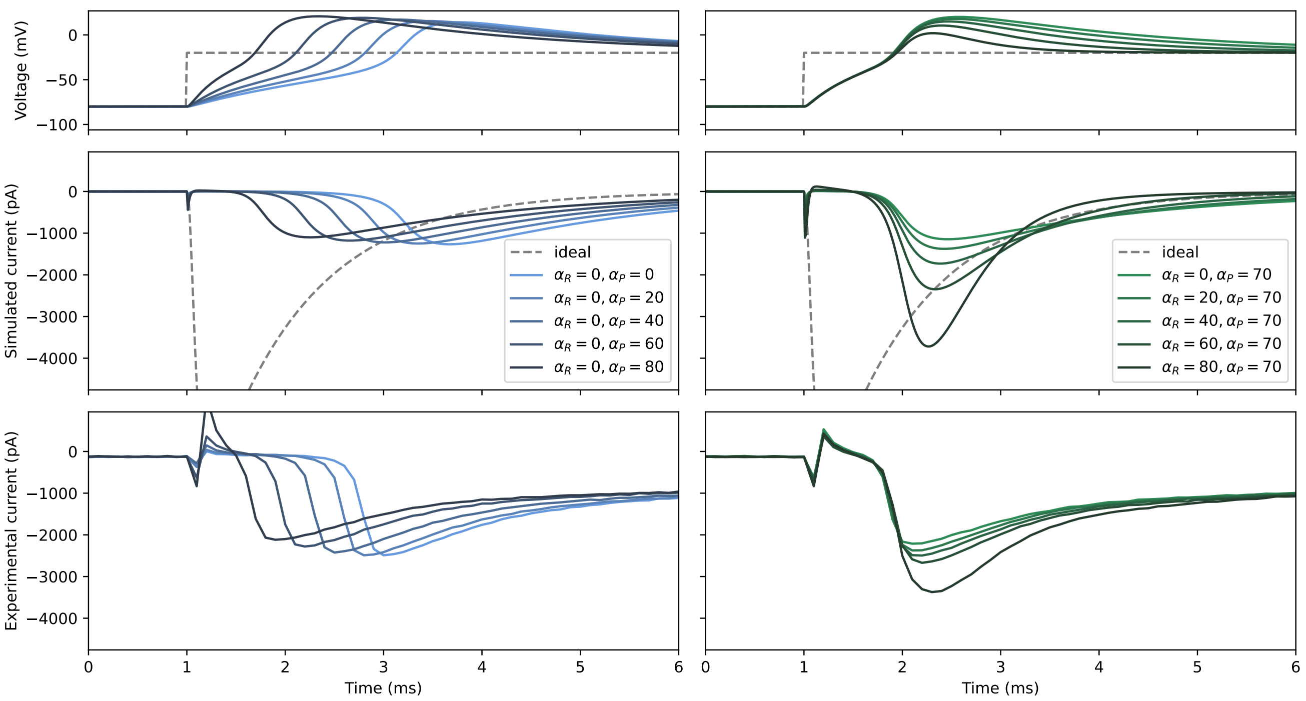

Figure S7: Forward sensitivity analysis of the experimental artifact of fast sodium current using Paci et al. (2020) and hiPSC-CMs (cell 3).

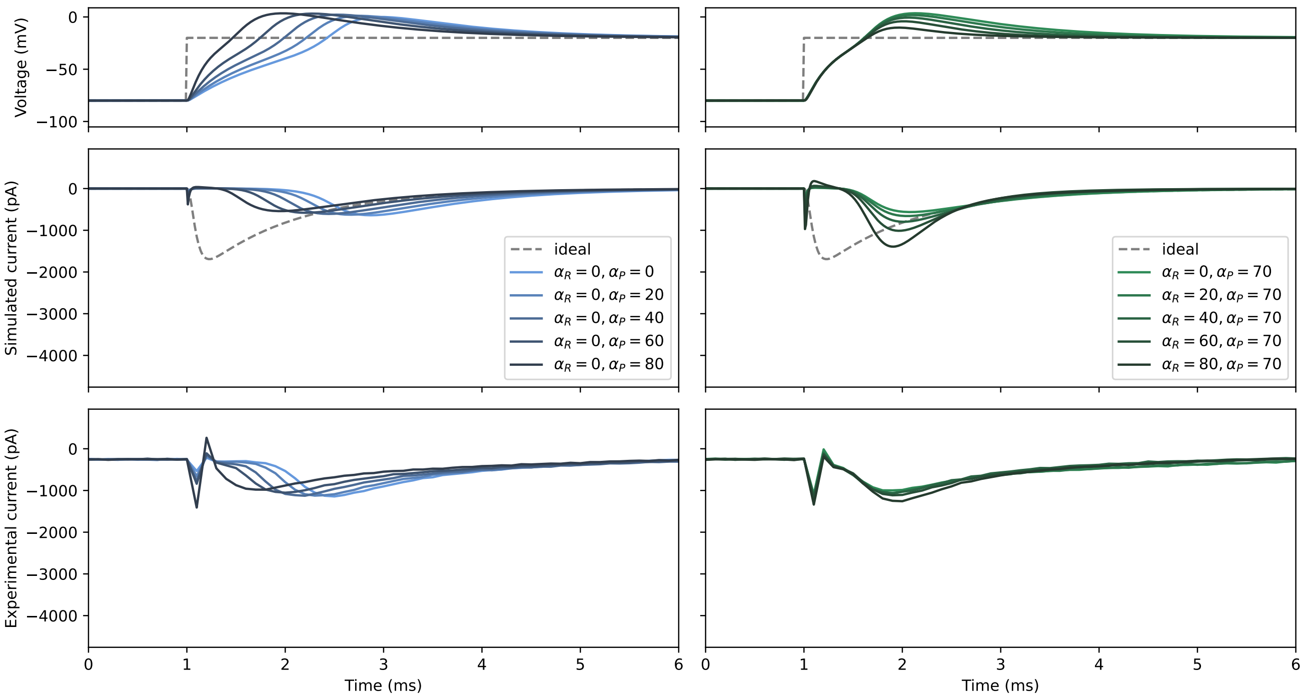

Figure S8: Forward sensitivity analysis of the experimental artifact of fast sodium current using Paci et al. (2020) and hiPSC-CMs (cell 4).

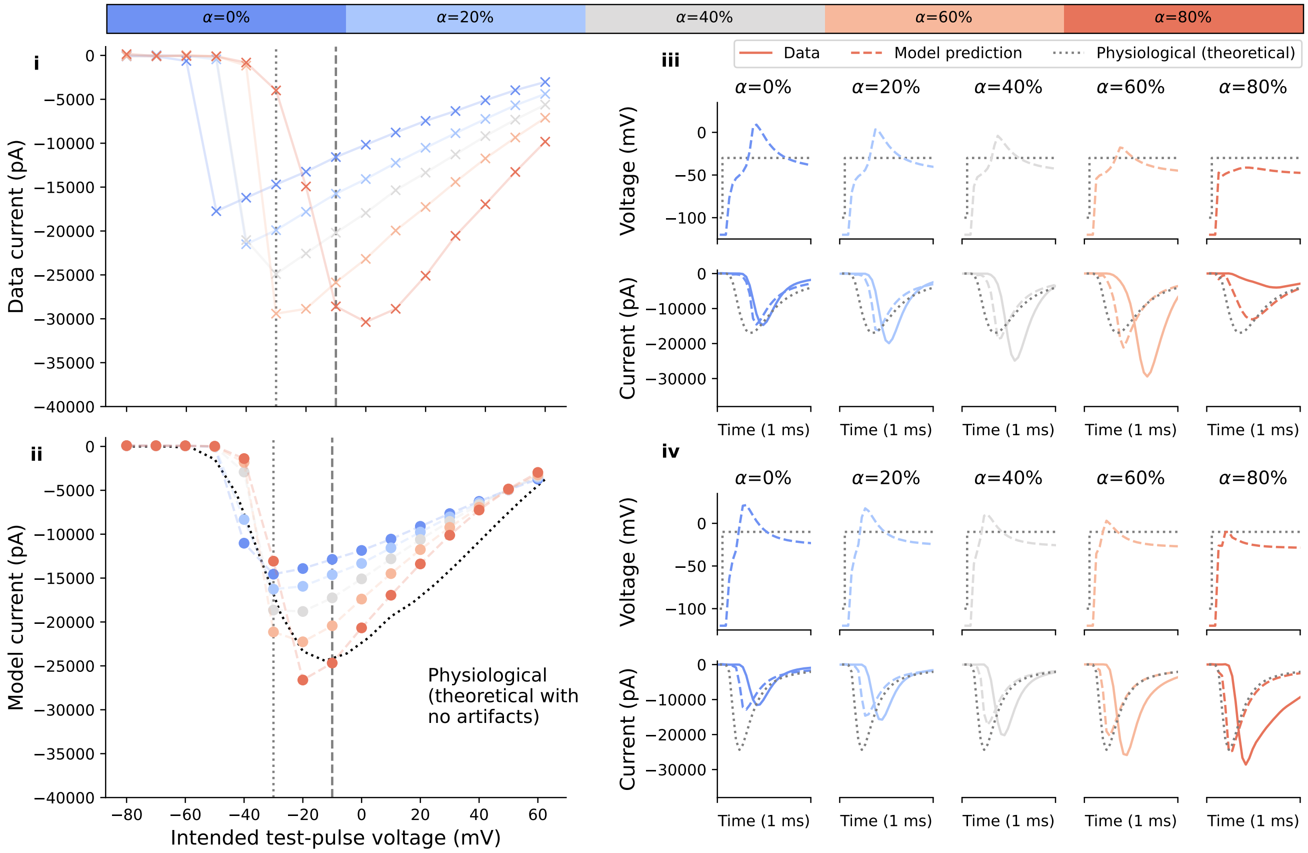

Figure S9: Correction of fast sodium current experiments, with estimated $R_\mathrm{s} = 4.3\,\mathrm{M\Omega}$ and $C_\mathrm{m} = 10.7\,\mathrm{pF}$ (cell 1) using the experimental artifact model.

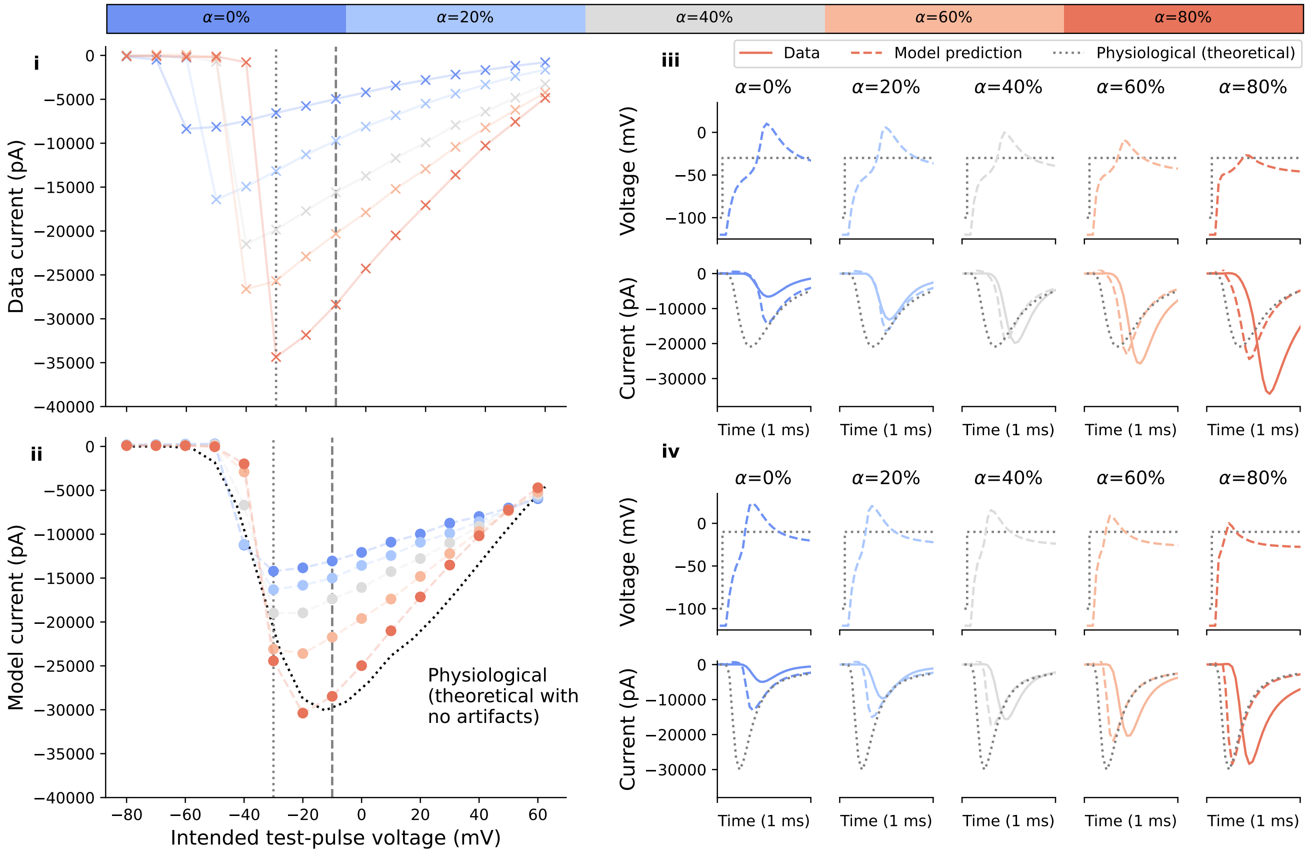

Figure S10: Correction of fast sodium current experiments, with estimated $R_\mathrm{s} = 4.1\,\mathrm{M\Omega}$ and $C_\mathrm{m} = 17.9\,\mathrm{pF}$ (cell 2) using the experimental artifact model.

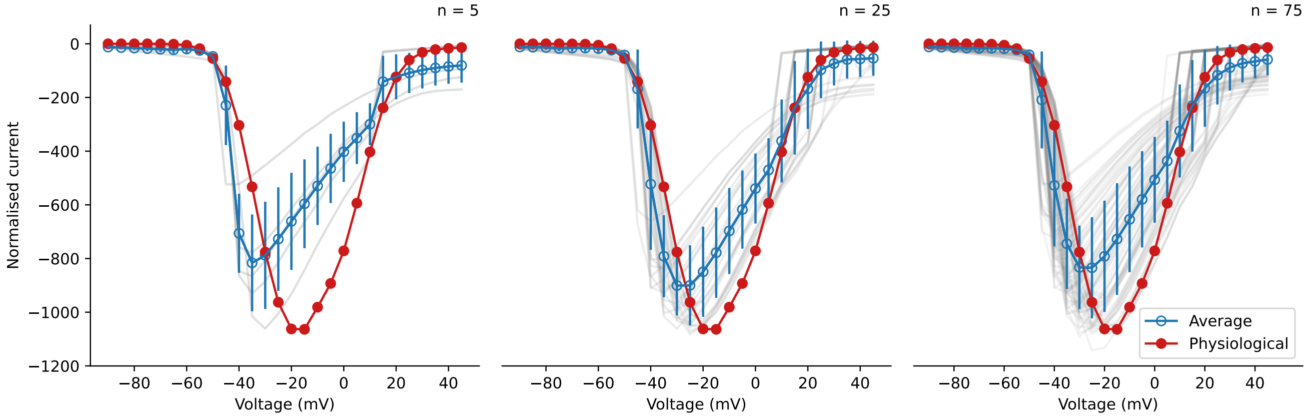

Figure S11: Consequences of averaging fast sodium current normalised I-V curves of multiple runs of experiments, with the average I-V curve shown in blue (averaged from transparent gray lines) and the artifact-free physiological I-V curve shown in red. Left to right shows different number of repeats, $n = 5$, 25, and 75, respectively. Error bars show the standard deviation (SD) of the data.

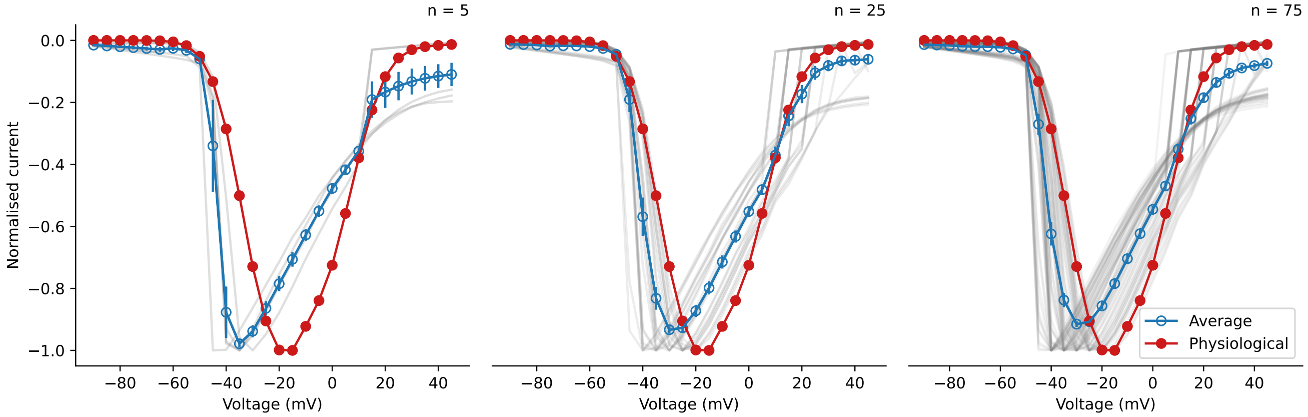

Figure S12: Consequences of averaging fast sodium current normalised I-V curves of multiple runs of experiments, with the average I-V curve shown in blue (averaged from transparent gray lines) and the artifact-free physiological I-V curve shown in red. Left to right shows different number of repeats, $n = 5$, 25, and 75, respectively. Error bars show the standard error of mean (SEM).

References

Axon Instruments Inc. (1999). Axopatch 200B patch clamp theory and operation. https://www.autom8.com/wp-content/uploads/2016/07/Axopatch-200B.pdf.

Gray, R. A. and Franz, M. R. (2020). A model for human action potential dynamics in vivo. *American Journal of Physiology-Heart and Circulatory Physiology*, 318(3):H534–H546.

HEKA Elektronik GmbH (2018). EPC 10 USB hardware manual version 2.8. http://www.heka.com/downloads/hardware/manual/m_epc10.pdf.

Horowitz, P., Hill, W., and Robinson, I. (2015). *The Art of Electronics*. Cambridge University Press, Cambridge, 3rd edition.

Lei, C. L. (2020). *Model-Driven Design and Uncertainty Quantification for Cardiac Electrophysiology Experiments*. PhD thesis, University of Oxford.

Lei, C. L., Clerx, M., Whittaker, D. G., Gavaghan, D. J., De Boer, T. P., and Mirams, G. R. (2020). Accounting for variability in ion current recordings using a mathematical model of artefacts in voltage-clamp experiments. *Philosophical Transactions of the Royal Society A*, 378(2173):20190348.

Neher, E. (1992). Correction for liquid junction potentials in patch clamp experiments. In *Methods in Enzymology*, volume 207 of *Ion Channels*, chapter 6, pages 123–131. Academic Press, Cambridge, MA.

Neher, E. (1995). Voltage offsets in patch-clamp experiments. In Sakmann, B. and Neher, E., editors, *Single-Channel Recording*, chapter 6, pages 147–153. Springer, Boston, MA, 2nd edition.

Paci, M., Passini, E., Klimas, A., Severi, S., Hyttinen, J., Rodriguez, B., and Entcheva, E. (2020). All-optical electrophysiology refines populations of in silico human iPSC-CMs for drug evaluation. *Biophysical Journal*, 118(10):2596–2611.

Sigworth, F. (1995a). Design of the EPC-9, a computer-controlled patch-clamp amplifier. 1. Hardware. *Journal of Neuroscience Methods*, 56(2):195–202.

Sigworth, F., Affolter, H., and Neher, E. (1995). Design of the EPC-9, a computer-controlled patch-clamp amplifier. 2. Software. *Journal of Neuroscience Methods*, 56(2):203–215.

Sigworth, F. J. (1995b). Electronic design of the patch clamp. In Sakmann, B. and Neher, E., editors, *Single-Channel Recording*, chapter 6, pages 95–127. Springer, Boston, MA, 2nd edition.

Weerakoon, P., Culurciello, E., Klemic, K. G., and Sigworth, F. J. (2009). An integrated patch-clamp potentiostat with electrode compensation. *IEEE Transactions on Biomedical Circuits and Systems*, 3(2):117–125.

Weerakoon, P., Culurciello, E., Yang, Y., Santos-Sacchi, J., Kindlmann, P. J., and Sigworth, F. J. (2010). Patch-clamp amplifiers on a chip. *Journal of Neuroscience Methods*, 192(2):187–192.
